# MHC-I genotype and tumor mutational burden predict response to immunotherapy

**DOI:** 10.1186/s13073-020-00743-4

**Published:** 2020-05-19

**Authors:** Aaron M. Goodman, Andrea Castro, Rachel Marty Pyke, Ryosuke Okamura, Shumei Kato, Paul Riviere, Garrett Frampton, Ethan Sokol, Xinlian Zhang, Edward D. Ball, Hannah Carter, Razelle Kurzrock

**Affiliations:** 1 Division of Blood and Marrow Transplantation, Department of Medicine, University of California San Diego, La Jolla, CA 92093 USA; 2 Division of Hematology/Oncology Center for Personalized Cancer Therapy, Department of Medicine, University of California San Diego, La Jolla, CA 92093 USA; 3 UC San Diego Moores Cancer Center, 855 Health Sciences Drive, La Jolla, CA 92093-0658 USA; 4 Division of Medical Genetics, Department of Medicine, University of California San Diego, La Jolla, CA 92093 USA; 5 Bioinformatics and Systems Biology Program, University of California San Diego, La Jolla, CA 92093 USA; 6 Health Science, Department of Biomedical Informatics, School of Medicine, University of California, San Diego, La Jolla, CA 92093 USA; 7 Foundation Medicine, Cambridge, MA 02141 USA; 8 Division of Biostatistics and Bioinformatics, Department of Family Medicine and Public Health, University of California San Diego, La Jolla, CA 92093 USA; 9 CIFAR, MaRS Centre, West Tower, 661 University Ave., Suite 505, Toronto, ON M5G 1M1 Canada

**Keywords:** MHC-I, TMB, Biomarkers, Checkpoint blockade

## Abstract

**Background:**

Immune checkpoint blockade (ICB) with antibodies inhibiting cytotoxic T lymphocyte-associated protein-4 (CTLA-4) and programmed cell death protein-1 (PD-1) (or its ligand (PD-L1)) can stimulate immune responses against cancer and have revolutionized the treatment of tumors. The influence of host germline genetics and its interaction with tumor neoantigens remains poorly defined. We sought to determine the interaction between tumor mutational burden (TMB) and the ability of a patient’s major histocompatibility complex class I (MHC-I) to efficiently present mutated driver neoantigens in predicting response ICB.

**Methods:**

Comprehensive genomic profiling was performed on 83 patients with diverse cancers treated with ICB to determine TMB and human leukocyte antigen-I (HLA-I) genotype. The ability of a patient’s MHC-I to efficiently present mutated driver neoantigens (defined by the Patient Harmonic-mean Best Rank (PHBR) score (with lower PHBR indicating more efficient presentation)) was calculated for each patient.

**Results:**

The median progression-free survival (PFS) for PHBR score < 0.5 vs. ≥ 0.5 was 5.1 vs. 4.4 months (*P* = 0.04). Using a TMB cutoff of 10 mutations/mb, the stable disease > 6 months/partial response/complete response rate, median PFS, and median overall survival (OS) of TMB high/PHBR high vs. TMB high/PHBR low were 43% vs. 78% (*P* = 0.049), 5.8 vs. 26.8 months (*P* = 0.03), and 17.2 months vs. not reached (*P* = 0.23), respectively. These findings were confirmed in an independent validation cohort of 32 patients.

**Conclusions:**

Poor presentation of driver mutation neoantigens by MHC-I may explain why some tumors (even with a high TMB) do not respond to ICB.

## Background

Immune checkpoint blockade (ICB) with antibodies inhibiting cytotoxic T lymphocyte-associated protein-4 (CTLA-4) and programmed cell death protein-1 (PD-1) (or its ligand (PD-L1)) can stimulate immune responses against cancer and has revolutionized the treatment of both solid [1] and hematologic malignancies [2]. Durable remissions after ICB have been reported in patients with diverse advanced cancers including, but not limited to, melanoma [3], non-small cell lung cancer (NSCLC) [4], renal cell carcinoma [5], and Hodgkin lymphoma [6]. Still, responses to ICB can be variable, toxicity can be serious, resistance is common [7], and hyperprogression can occur [8]. Further, the majority of patients will not benefit from ICB, and there is a need to better select patients for treatment [9].

Multiple factors influence the immune response against tumors including tumor T cell infiltration, tumor mutational burden (TMB), PD-L1 expression, interferon signaling, mismatch repair (MMR) deficiency, tumor aneuploidy, and possibly the intestinal microbiota [10]. Biomarkers that have entered clinical practice include PD-L1 expression measured by immunohistochemistry (IHC) [11], *PD-L1* amplification [12], microsatellite instability (MSI) [13, 14], and TMB [15, 16].

Somatic mutations in tumors can be recognized by the immune system [17] resulting in tumor eradication. MMR-deficient/MSI-high tumors have 10 to 100 times as many somatic alterations as MMR-proficient tumors [13], resulting in exquisite sensitivity to ICB therapy [14]. Most cancers harboring MMR alterations are associated with high TMB [18]. In addition, many cancers harbor high TMB (10–20% depending on the definition of high TMB), even without MMR alterations [15, 19]. Higher TMB correlates with better treatment outcomes, including higher response rates and longer progression-free survival (PFS) and overall survival (OS), in diverse cancers treated with immunotherapies [15].

Despite the improved efficacy of ICB in TMB-high tumors, approximately 40–60% of patients with a high TMB will not respond [15, 16]. To date, there is no sufficient way to predict which patients with high TMB will or will not respond to ICB. It has been hypothesized that tumors with high TMB and low PD-L1 expression might not respond as well to ICB; however, studies have demonstrated higher response rates and PFS in patients with high TMB versus low TMB, irrespective of PD-L1 expression [20].

Major histocompatibility complex class I (MHC-I) molecules, encoded by the human leukocyte antigen-I (HLA-I) locus, present intracellular peptides on the surface of both normal and tumor cells for recognition by CD8+ cytotoxic T cells [21]. HLA-I genotype has been linked to a variety of different immune responses including infection [22], autoimmune diseases [23], and the graft versus host/tumor effect seen after allogeneic stem cell transplantation [24]. There is accumulating experimental evidence suggesting that immunosurveillance shapes the mutational landscapes of cancers through the elimination of early tumor cells [25–27]. In addition, the predicted number of MHC-I-associated neoantigens has been shown to be low in certain tumors suggesting immune-mediated elimination [28], and the anti-tumor activity of ICB is dependent on MHC-I presentation of specific tumor-derived peptides [29, 30].

Marty et al. developed a residue-centric patient MHC-I presentation score (termed the Patient Harmonic-mean Best Rank (PHBR) score) that describes a person’s ability to present specific cancer mutations to CD8+ T cells, and found that PHBR scores correlated with the likelihood of mutations to emerge in a patient’s tumor [31]. Poor presentation of a mutation across patients was correlated with higher frequency among tumors. These results support that MHC-I genotype-restricted immunoediting shapes the mutational landscape of malignancies.

It has been suggested that the presence of a high-quality neoantigen is required for response to therapy [32] while a high burden of neoantigens has been associated with impaired anti-tumor immune activity [33]; thus, we focused on neoantigen quality over quantity by using patient minimum PHBR score (i.e., best-presented mutation) to predict whether mutations observed in a patient’s tumor are likely to generate effectively presented neoantigens. We assessed the ability of PHBR and TMB to predict response to ICB in diverse solid tumors.

## Methods

### Patient selection

Three hundred and twenty-eight patients with diverse solid tumors treated with ICB (4/2010–5/2018) at a single institution were reviewed. Patients with melanoma, tumors that were not sequenced by Foundation Medicine (FM), and patients without an identified missense alteration by NGS were excluded. We excluded patients without next-generation sequencing or those with sequencing, but no identified missense alterations, because PHBR cannot be calculated in those cases; we omitted melanoma because melanoma patients have disproportionately high TMBs and high response rates to immunotherapy as compared to the majority of other cancers. All patients were treated with anti-PD-1/L1 monotherapy (or in combination with a second agent). The validation cohort was composed of thirty-two NSCLC patients treated with pembrolizumab (starting from 2012 to 2013) at Memorial Sloan Kettering and the University of California Los Angeles. All validation patients had consented to Institutional Review Board-approved protocols regarding tissue collection and sequencing.

### TMB and HLA-I sequencing

Patients had NGS performed on tumor samples to determine genetic alterations, TMB, and HLA-I genotype [34]. Formalin-fixed paraffin-embedded tumor samples were submitted for NGS to FM [clinical laboratory improvement amendments (CLIA)-certified lab]. The FoundationOne assay was used (hybrid-capture-based NGS; 236 or 315 genes; http://www.foundationone.com/). The methods have been previously described [34]. Average sequencing depth of coverage was greater than 250X, with > 100X at > 99% of exons. For TMB, the number of somatic mutations detected on NGS (interrogating 1.2 mb of the genome) is quantified and that value extrapolated to the whole exome using a validated algorithm [35]. Alterations likely or known to be bona fide oncogenic drivers and germline polymorphisms are excluded. TMB was measured in mutations per megabase (mb). Sequence-derived HLA-A/B/C typing was conducted by back-converting BAM files to fastq, then performing HLA realignment and typing using OptiType [36].

### PHBR

The Patient Harmonic-mean Best Rank (PHBR) score as previously described [31], is a metric that represents how well the specific HLA-I genotype of an individual can bind and present a specific missense mutation. Each patient was assigned the PHBR score of his or her best-presented missense driver mutation. For patients with two or more missense mutations, only the mutation with the lowest PHBR score was selected. PHBR low (strong presentation) and high (poor presentation) were defined as < 0.5 and ≥ 0.5, respectively.

### Mapping Foundation Medicine mutations to peptides

RefSeq transcript IDs from the FM variant spreadsheet were mapped to corresponding Ensembl transcript IDs with coding (CDS) sequences. For evaluation of missense mutations, we replaced the native amino acid residue with the mutated residue and selected all 38 possible peptides of length 8–11 that covered the mutated amino acid residue. For evaluation of in-frame insertion and deletion mutations, bases were inserted or deleted from the CDS sequence according to the “cds effect” column from the FM data. The new CDS sequence was then translated into an amino acid sequence using the Seq.translate function from Biopython (Bio) package [37]. We then selected any resulting novel peptides of length 8–11 for affinity analysis.

### Affinity analysis

We calculated the allele-specific binding affinities of the previously described mutated peptides using NetMHCpan4.0 [38]. Conventionally, a NetMHCpan4.0 binding affinity percentile rank less than 2 indicates weak peptide-MHC binding, while a binding affinity percentile rank less than 0.5 indicates strong peptide-MHC binding [39]. Patient Harmonic-mean Best Rank PHBR scores [31] were used to represent a patient’s ability to present the mutations in their tumor. *HLA-A*, *HLA-B*, and *HLA-C* alleles were obtained from FM. We evaluated the binding affinity of each HLA allele for 38 possible peptides of length 8–11 overlapping each mutation using NetMHCpan4.0. For individual alleles, the best rank percentile from NetMHCpan4.0 out of the 38 possible peptides was assigned. Best rank percentiles for all 6 alleles were aggregated into the PHBR score using a harmonic mean. High PHBR scores are indicative of poor affinity of peptides overlapping a mutation with the patient’s MHC-I molecules and vice versa.

### Validation

Matched tumor-normal exome sequencing fastq files obtained from [40] (dbGaP study accession phs000980.v1.p1.c1) were preprocessed and mutations called according to the GATK best practice workflow. Only mutations occurring in the 309 genes from the Foundation Medicine gene panel were retained. HLA typing was done in silico using the OptiType software package [41]. Mutated peptides were created using the same method as described above. Similarly, PHBR scores were generated as described previously.

### Statistical analysis

We used the Fisher exact test to assess categorical variables. *P* values < 0.05 were considered significant (values < 0.10 were included in the multivariable regression analyses). Overall benefit rate (OBR) (stable disease for ≥ 6 months and partial or complete response) was determined (RECIST criteria). Median PFS and OS were calculated from the start of checkpoint blockade and data was censored at the last visit for patients still progression free or alive, respectively, for PFS and OS. For the outcome analysis, comparisons were made between TMB low vs. high and PHBR low vs. high. Patients with no TMB values were assigned to the low TMB category for discrete analyses, and a pseudocount of 0.001 was added to TMB for all patients. We performed a Cox proportional hazards regression stratified by high (≥ 10 mutations/mb) or low (< 10 mutations/mb) TMB to quantify the specific effect of PHBR on PFS. These findings were visualized using Kaplan-Meier curves. Statistical analysis was performed on R version 3.5.2 and IBM SPSS Statistics version 24.

## Results

Eighty-three patients with 20 different solid malignancies were identified (Table 1, Additional file 1: Table S1, and Additional file 1: Fig. S1, and Additional file 1: Fig. S2). The most common malignancies in the cohort included non-small cell lung cancer (NSCLC) (*N* = 26), cutaneous squamous cell carcinoma (SCC) (*N* = 10), and head and neck SCC (*N* = 9). Sixty-six patients were treated with PD-1/L1 monotherapy and 17 with combination therapy. The OBR (stable disease (SD) ≥ 6 months/partial and complete response (PR/CR)) was 43%. Thirty-two patients had at least one PHBR score of < 0.5 and fifty-one patients had a minimum score ≥ 0.5 (lower scores reflecting better neoantigen presentation) (Additional file 1: Fig. S3). A minimum PHBR score ≥ 0.5 was significantly associated in univariate analysis with progressive disease (*P* = 0.02), non-cutaneous SCC malignancies (*P* = 0.04), and a TMB < 50 mutations/mb (*P* = 0.05).

**Table 1:** Patient demographics by PHBR score (< 0.5 vs. ≥ 0.5) (*N* = 83)

Variable	Group	*N* (82)	PHBR < 0.5 (*N* = 32)	PHBR ≥ 0.5 (*N* = 51)	Relative risk (95% CI)^1^	*P* value^2^
Sex	Male	46	22 (48%)	24 (52%)	1.77 (0.96–3.26)	0.07
	Female	37	10 (27%)	27 (73%)		
Ethnicity	Caucasian	71	27 (38%)	44 (62%)	0.91 (0.44–1.90)	> 0.99
	Others^3^	12	5 (42%)	7 (58%)		
Age^4^ (years)	< 60	17	6 (35%)	11 (65%)	0.90 (0.44–1.82)	> 0.99
	≥ 60	66	26 (39%)	40 (61%)		
Tumor type	Head and neck SCC	9	4 (44%)	5 (56%)	1.18 (0.54–2.58)	0.73
	Others	74	28 (38%)	46 (62%)		
	NSCLC	26	7 (27%)	19 (73%)	0.61 (0.31–1.23)	0.16
	Others	57	25 (44%)	32 (56%)		
	Cutaneous SCC	10	7 (70%)	3 (30%)	2.04 (1.22–3.42)	**0.04**
	Others	73	26 (34%)	48 (66%)		
	Others^5^	38	14 (37%)	24 (63%)	0.92 (0.53–1.60)	0.82
	Head and neck SCC, NSCLC, and cutaneous SCC	45	18 (40%)	27 (60%)		
TMB^6^ (mutations/mb)	< 50	65	21 (32%)	44 (68%)	0.49 (0.28–0.83)	**0.048**
	≥ 50	12	8 (67%)	4 (33%)		
	< 20	56	18 (32%)	38 (68%)	0.61 (0.35–1.07)	0.12
	≥ 20	21	11 (52%)	10 (48%)		
	< 10	38	11 (29%)	27 (71%)	0.63 (0.34–1.15)	0.16
	≥ 10	39	18 (46%)	21 (54%)		
PD-1/L1 Therapy	Monotherapy	66	26 (39%)	40 (61%)	1.12 (0.55–2.27)	> 0.99
	Combination	17	6 (35%)	11 (65%)		
Overall benefit rate	SD ≥ 6 months/PR/CR^7^	36	17 (47%)	19 (53%)	1.45 (0.84–2.49)	0.25
	Others	46	15 (33%)	31 (67%)		
	PD	32	7 (22%)	25 (78%)	0.45 (0.22–0.91)	**0.02**
	Others	51	25 (49%)	26 (51%)		

^1^Relative risk for PHBR < 0.5

^2^Calculated using Fisher’s exact test

^3^Others: African American (*N* = 2), Asian (*N* = 4), Hispanic (*N* = 5), and unknown (*N* = 1)

^4^At time of initiation of treatment with immunotherapy

^5^Others: adrenal (*N* = 1), appendix (*N* = 4), basal cell carcinoma (*N* = 3), breast cancer (*N* = 6), cervical (*N* = 1), cholangiocarcinoma (*N* = 1), colorectal (*N* = 2), duodenal (*N* = 1), gastroesophageal (*N* = 5), glioblastoma (*N* = 2), thyroid (*N* = 1), prostate (*N* = 1), rectal squamous cell carcinoma (*N* = 1), renal cell carcinoma (*N* = 1), sarcoma (*N* = 3), urothelial (*N* = 4), and urethral squamous cell carcinoma (*N* = 1)

^6^TMB was performed on 77 patients

^7^One patient had SD, but had not reached to 6 months. Only 82 patients were evaluable for this comparison

*Abbreviations*: *CR* complete response, *HR* hazard ratio, *NR* not reached to 50%, *NSCLC* non-small cell lung cancer, *OS* overall survival, *PFS* progression-free survival, *PD* progressive disease, *PHBR* Patient Harmonic-mean Best Rank, *PR* partial response, *RR* relative risk, *SC*C squamous cell carcinoma, *SD* stable disease, *TMB* tumor mutational burden

**Fig. 1:**
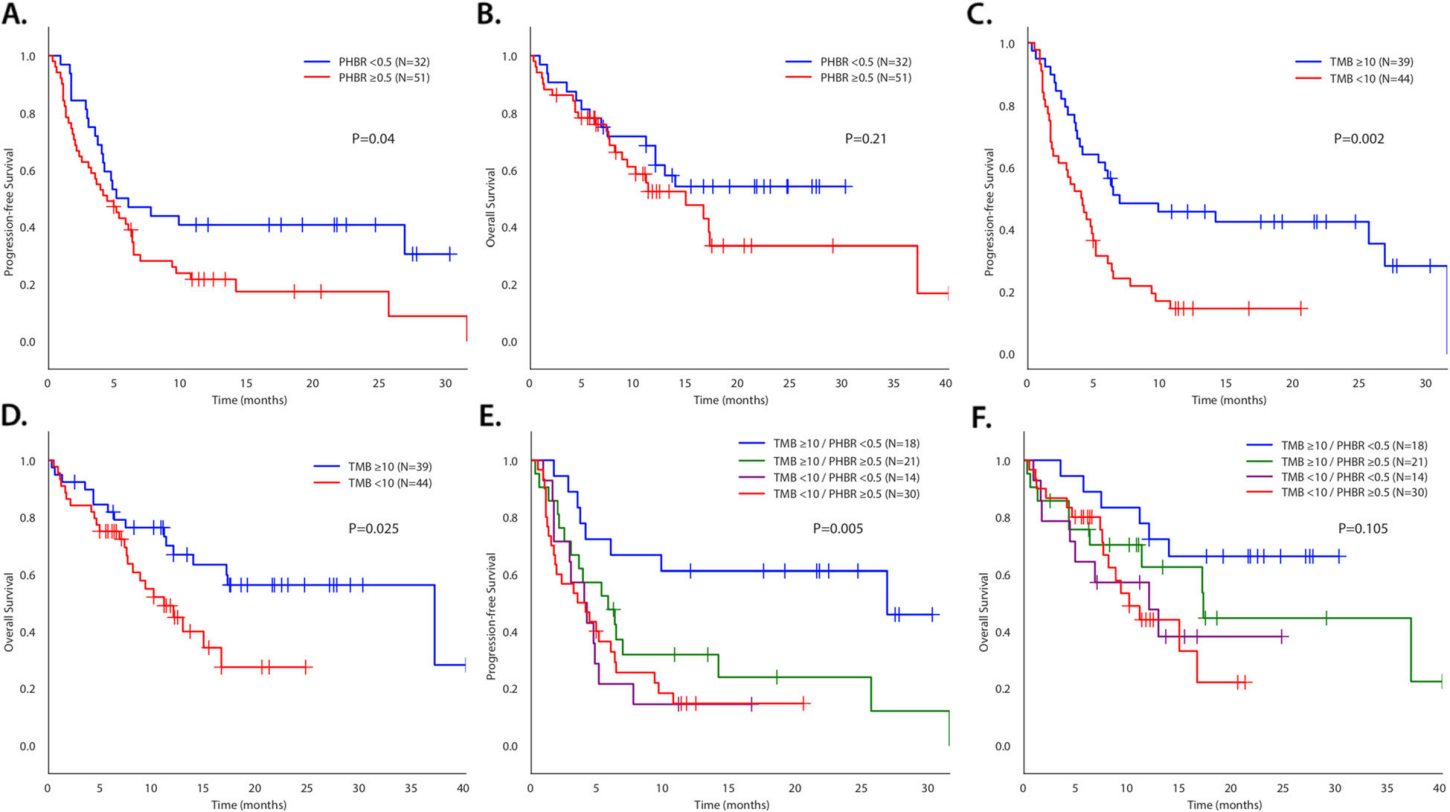
Kaplan-Meier PFS and OS for patients treated with immunotherapy. *P* values in Fig. 1 compare all four categories. They differ slightly from *P* values in Table 3, which compares value to the reference. PFS (**a**) and OS (**b**) dichotomized by PHBR < 0.5 and ≥ 0.5 (*N* = 83). PFS (**c**) and OS (**d**) dichotomized by TMB < 10 and ≥ 10 mutations/mb (*N* = 83). PFS (**e**) and OS (**f**) separated by TMB < 10 and ≥ 10 and PHBR < 0.5 and ≥ 0.5 (*N* = 83). For PFS (**e**), *P* = 0.005 for difference between all four curves. Curve for TMB ≥ 10/PHBR < 0.5 versus TMB ≥ 10/PHBR ≥ 0.5 was significantly different (*P* = 0.025); TMB ≥ 10/PHBR ≥ 0.5 did not differ significantly from TMB < 10/PHBR ≥ 0.5 (*P* = 0.19) or from TMB < 10/PHBR < 0.5 (*P* = 0.26); TMB < 10/PHBR ≥ 0.5 did not differ significantly from TMB < 10/PHBR < 0.5 (*P* = 0.91). For OS (**f**), *P* = 0.1 for difference between all four curves. Differences between individual curves were not statistically different

In univariate analysis (Table 2), only higher TMB (≥ 10 mutations/mb) was associated with a better OBR. Caucasian ethnicity, high TMB, and a minimum PHBR score < 0.5 were all significantly associated with longer median PFS while male sex, Caucasian ethnicity, and high TMB were associated with longer median OS. The median PFS for low versus high PHBR scores was 5.1 vs. 4.4 months (*P* = 0.04) (Fig. 1). The median PFS for high versus low TMB at various thresholds (10, 20, 50) was 6.9 vs. 4.0 months (*P* = 0.001), 14.1 vs. 4.2 months (*P* = 0.01), and 26.8 vs. 4.4 months (*P* = 0.03), respectively.

**Table 2:** Univariate analysis of factors affecting outcome for patients treated with immune checkpoint blockade (*N* = 83)

	Rate of SD ≥ 6 month/PR/CR^1^		PFS			OS		
Variable	***N*** (%)	***P*** value^2^	Median, months	HR (95% CI)	***P*** value^3^	Median, months	HR (95% CI)	***P*** value^3^
**Sex**								
Male (*N* = 46) vs. female (*N* = 37)	23 (51%) vs. 13 (35%)	0.18	6.3 vs. 4.1	0.63 (0.38–1.04)	0.07	NR (MFU, 19.1) vs. 12.0	0.51 (0.27–0.95)	**0.03**
**Ethnicity**								
Caucasian (*N* = 71) vs. others^4^ (*N* = 12)	32 (45%) vs. 4 (36%)	0.75	4.9 vs. 2.9	0.52 (0.26–1.00)	**0.045**	18.5 vs. 8.2	0.45 (0.19–1.06)	**0.004**
**Age**^5^, years								
< 60 (*N* = 17) vs. ≥ 60 (*N* = 66)	6 (35%) vs. 30 (46%)	0.58	3.5 vs. 5.1	1.29 (0.70–2.39)	0.41	12.0 vs. 14.9	0.86 (0.36–2.06)	0.73
**Tumor type**								
Head and neck SCC (*N* = 9) vs. not (*N* = 74)	4 (44%) vs. 32 (44%)	> 0.99	4.8 vs. 4.9	1.01 (0.46–2.22)	0.99	12.9 vs. 16.6	1.11 (0.43–2.84)	0.83
NSCLC (*N* = 26) vs. not (*N* = 57)	8 (31%) vs. 28 (50%)	0.15	3.0 vs. 6.0	1.67 (0.99–2.81)	**0.05**	9.3 vs. 16.6	1.37 (0.71–2.64)	0.34
Cutaneous SCC (*N* = 10) vs. not (*N* = 73)	7 (70%) vs. 29 (40%)	0.10	26.8 vs. 4.7	0.43 (0.17–1.08)	0.06	NR (median follow-up, 21.7) vs. 13.9 14.9 vs. 17.1	0.43 (0.13–1.40)	0.15
Others^6^ (*N* = 38) vs. head and neck SCC, NSCLC, and cutaneous SCC (*N* = 45)	17 (46%) vs. 19 (42%)	0.82	5.1 vs. 4.8	0.91 (0.55–1.52)	0.72		1.02 (0.54–1.93)	0.95
**TMB**^7^, mutations/mb								
≥ 50 (*N* = 12) vs. < 50 (*N* = 65)	9 (75%) vs. 25 (39%)	**0.03**	26.8 vs. 4.4	0.40 (0.17–0.94)	**0.03**	NR (median follow-up, 17.5) vs. 12.9	0.39 (0.12–1.27)	0.10
≥ 20 (*N* = 21) vs. < 20 (*N* = 56)	14 (67%) vs. 20 (36%)	**0.02**	14.1 vs. 4.2	0.45 (0.23–0.85)	**0.01**	NR (median follow-up, 22.4) vs. 12.0	0.42 (0.19–0.96)	**0.03**
≥ 10 (*N* = 39) vs. < 10 (N = 38)	23 (59%) vs. 11 (29%)	**0.01**	6.9 vs. 4.0	0.40 (0.23–0.68)	**0.001**	37.1 vs. 10.1	0.42 (0.21–0.82)	**0.009**
**PHBR**								
< 0.5 (*N* = 32) vs. ≥ 0.5 (N = 51)	17 (53%) vs. 19 (38%)	0.25	5.1 vs. 4.4	0.58 (0.34–0.99)	**0.04**	NR (median follow-up, 21.7) vs. 14.9	0.66 (0.34–1.27)	0.21
**PD-1/L1 therapy**								
Monotherapy (*N* = 66) vs. combination (*N* = 17)	25 (39%) vs. 11 (65%)	0.06	4.1 vs. 6.3	1.17 (0.63–2.16)	0.63	17.1 vs. 11.3	0.78 (0.37–1.66)	0.51

^1^Thirty-six patients achieved SD with ≥ 6 months/PR/CR. One patient attained ongoing SD, but has not yet reached 6-month follow-up and is therefore not considered evaluable for this parameter; only 82 patients were evaluable for this comparison

^2^Calculated using Fisher’s exact test

^3^Calculated using the log-rank test

^4^Others: African American (*N* = 2), Asian (*N* = 4), Hispanic (*N* = 5), and unknown (*N* = 1)

^5^At time of initiation of treatment with immunotherapy

^6^Others: adrenal (*N* = 1), appendix (*N* = 4), basal cell carcinoma (*N* = 3), breast cancer (*N* = 6), cervical (*N* = 1), cholangiocarcinoma (*N* = 1), colorectal (*N* = 2), duodenal (*N* = 1), gastroesophageal (*N* = 5), glioblastoma (*N* = 2), thyroid (*N* = 1), prostate (*N* = 1), rectal squamous cell carcinoma (*N* = 1), renal cell carcinoma (*N* = 1), sarcoma (*N* = 3), urothelial (*N* = 4), and urethral squamous cell carcinoma (*N* = 1)

^7^Seventy-seven patients with TMB were evaluable for the response rate, PFS, and OS

*Abbreviations*: *HR* hazard ratio, *NR* not reached to 50%, *NSCLC* non-small cell lung cancer, *OS* overall survival, *PFS* progression-free survival, *PHBR* Patient Harmonic-mean Best Rank, *SCC* squamous cell carcinoma, *TMB* tumor mutational burden

Using a TMB cutoff of 10 mutations/mb, the OBR, median PFS, and median OS of TMB low/PHBR high vs. TMB high/PHBR low were 33% vs. 78% (*P* = 0.006), 3.5 vs. 26.8 months (*P* < 0.001), and 10.1 months vs. not reached (*P* = 0.008), respectively (Fig. 1 and Table 3). Results remain when we exclude patients who had unknown TMB values (Additional file 1: Fig. S4). Patients with high TMB had greater OBR (43% vs. 78%, *P* = 0.049), greater PFS (5.8 vs. 26.8 months, *P* = 0.03), and greater median overall survival (17.2 months vs. not reached, *P* = 0.23) when accompanied by a well-presented mutation (low PHBR) than their counterparts with less well-presented mutations (high PHBR) (Table 3, Additional file 1: Fig. S5).

**Table 3:** Overall response rate, PFS, and OS segregated by TMB low/high and PHBR low/high among patients treated with immunotherapy patients (*N* = 77 with TMB available)

	Rate of SD with ≥ 6 month/PR/CR^1^		PFS			OS		
Group	***N*** (%)	***P*** value	Median (months)	HR (95% CI)	***P*** value^2^	Median (months)	HR (95% CI)	***P*** value^2^
**TMB/PHBR** (TMB cutoff = 10 mutations/mb)								
Low/high (*N* = 27) vs. low/low (*N* = 11)	9 (33%) vs. 2 (18%)	0.45	3.5 vs. 4.2	1.01 (0.48–2.12)	0.99	10.1 vs. 12.0	0.90 (0.37–2.22)	0.82
Low/high (*N* = 27) vs. high/high (*N* = 21)	9 (33%) vs. 9 (43%)	0.56	3.5 vs. 5.8	0.76 (0.54–1.05)	0.09	10.1 vs. 17.2	0.72 (0.47–1.10)	0.12
Low/high (*N* = 27) vs. high/low (*N* = 18)	9 (33%) vs. 14 (78%)	**0.006**	3.5 vs. 26.8	0.62 (0.47–0.83)	**< 0.001**	10.1 vs. NR^3^	0.66 (0.47–0.91)	**0.008**
Low/low (*N* = 11) vs. high/high (*N* = 21)	2 (18%) vs. 9 (43%)	0.25	4.2 vs. 5.8	0.58 (0.25–1.31)	0.18	12.0 vs. 17.2	0.62 (0.23–1.69)	0.34
Low/low (*N* = 11) vs. high/low (*N* = 18)	2 (18%) vs. 14 (78%)	**0.003**	4.2 vs. 26.8	0.50 (0.30–0.83)	**0.003**	12.0 vs. NR^3^	0.59 (0.34–1.02)	**0.049**
High/high (*N* = 21) vs. high/low (*N* = 18)	9 (43%) vs. 14 (78%)	**0.049**	5.8 vs. 26.8	0.39 (0.16–0.91)	**0.03**	17.2 vs. NR^3^	0.53 (0.19–1.50)	0.23
**TMB/PHBR** (TMB cutoff = 20 mutations/mb)								
Low/high (*N* = 38) vs. low/low (*N* = 18)	13 (34%) vs. 7 (39%)	0.77	4.1 vs. 4.2	0.89 (0.47–1.67)	0.71	11.1 vs. 12.0	0.81 (0.38–1.73)	0.58
Low/high (*N* = 38) vs. high/high (*N* = 10)	13 (34%) vs. 5 (50%)	0.47	4.1 vs. 3.6	0.96 (0.65–1.41)	0.82	11.1 vs. 17.2	0.76 (0.45–1.31)	0.32
Low/high (*N* = 38) vs. high/low (*N* = 11)	13 (34%) vs. 9 (82%)	**0.007**	4.1 vs. NR^4^	0.59 (0.41–0.84)	**0.001**	11.1 vs. NR^5^	0.66 (0.44–0.99)	**0.03**
Low/low (*N* = 18) vs. high/high (*N* = 10)	7 (39%) vs. 5 (50%)	0.70	4.2 vs. 3.6	1.06 (0.44–2.53)	0.90	12.0 vs. 17.2	0.82 (0.26–2.62)	0.74
Low/low (*N* = 18) vs. high/low (*N* = 11)	7 (39%) vs. 9 (82%)	0.052	4.2 vs. NR^4^	0.46 (0.24–0.86)	**0.007**	12.0 vs. NR^5^	0.60 (0.31–1.15)	0.11
High/high (*N* = 10) vs. high/low (*N* = 11)	5 (50%) vs. 9 (82%)	0.18	3.6 vs. NR^4^	0.16 (0.04–0.64)	**0.004**	17.2 vs. NR^5^	0.37 (0.08–1.70)	0.19

^1^Thirty-six patients achieved SD with ≥ 6 month/PR/CR

^2^*P* values in Fig. 1 are different as they compare all four categories at the same time

^3^Not reached to the median (median follow-up duration, 23.0 months)

^4^Not reached to the median (median follow-up duration, 24.6 months)

^5^Not reached to the median (median follow-up duration, 27.0 months)

*Abbreviations*: *HR* hazard ratio, *NR* not reached to 50%, *OS* overall survival, *PFS* progression-free survival, *PHBR* Patient Harmonic-mean Best Rank, *TMB* tumor mutational burden

In a multivariable regression analysis (Table 4) of factors affecting outcome for patients treated with immunotherapy, high TMB (*P* = 0.01) and treatment with combination therapy (*P* = 0.006) were significantly associated with a higher OBR. Only high TMB was significantly associated with a prolonged median PFS (*P* = 0.01) and OS (*P* = 0.04). However, in stratified Cox regression, which allows for different hazard functions among strata [42] of PHBR in the higher TMB (≥ 10 mutations/mb) patients (*N* = 39), we found that a low PHBR score is significantly predictive of PFS (HR 0.39 (0.16–0.91), *P* = 0.03). Multivariable regression analysis in this cohort of 39 patients with high TMB showed that PHBR, but not TMB, was selected as an independent factor predicting both OBR and longer PFS (*P* = 0.049 and 0.03, respectively) (Additional file 1: Table S2 and Table S3). In contrast, PHBR had no effect on PFS (*P* = 0.98) in patients with lower TMB (< 10 mutations/mb) (*N* = 38). Plotting Kaplan-Meier curves of patients based on lower or higher TMB and low or high PHBR found similar results in the general cohort (i.e., PHBR low versus high is associated with significant separation of the curves in patients with TMB ≥ 10 mutations/mb, but not in patients with lower TMBs (Fig. 1)). Finally, overall, Spearman correlation coefficient between TMB and PHBR was 0.31 with a *P* value of 0.01, consistent with a higher likelihood of carrying a low PHBR mutation when TMB is high (Additional file 1: Fig. S6).

**Table 4:** Multivariable regression analysis of factors affecting outcome for patients treated with immunotherapy (*N* = 77 with TMB available)

Group	OR (95% CI)	***P*** value
**Rate of SD ≥ 6 month/PR/CR**		
Cutaneous SCC versus others	3.96 (0.69–22.64)	0.12
TMB ≥ 10 mutations/mb versus < 10	4.51 (1.40–14.61)	**0.01**
PD-1/L1 monotherapy versus combination	0.15 (0.04–0.58)	**0.006**
**Progression-free survival**		
Male versus female	0.94 (0.53–1.68)	0.83
Caucasian versus others	0.69 (0.33–1.43)	0.32
NSCLC versus others	1.52 (0.86–2.67)	0.15
Cutaneous SCC versus others	0.71 (0.22–2.26)	0.56
TMB ≥ 10 mutations/mb versus others	0.47 (0.26–0.86)	**0.01**
PHBR < 0.5 versus ≥ 0.5	0.75 (0.41–1.38)	0.36
**Overall survival**		
Male versus female	0.64 (0.33–1.26)	0.20
Caucasian versus others	0.68 (0.27–1.72)	0.42
TMB ≥ 10 mutations/mb versus < 10	0.48 (0.24–0.970)	**0.04**

Variables with *P* value of ≤ 0.1 in univariate (Table 2) were included in the multivariable regression analysis

*Abbreviations*: *CR* complete response, *HR* hazard ratio, *NSCLC* non-small cell lung cancer, *OR* odds ratio, *PHBR* Patient Harmonic-mean Best Rank, *PR* partial response, *SCC* squamous cell carcinoma, *SD* stable disease, *TMB* tumor mutational burden

Next, we evaluated the added value of PHBR with respect to TMB from another perspective. We first fit a logistic regression model relating OBR to all potential confounders, using a backward selection process where we removed confounders one at a time and compared models using Akaike Information Criterion (AIC) scores [43]. We kept all confounders for which exclusion did not result in an increased AIC (i.e., the model better explained the data when the confounder was included). The retained confounders included MSI status, ethnicity, and the type of cancer each patient was diagnosed with. Then, we sequentially added TMB and PHBR to the regression model, using AIC once again to compare models (Table S8). We found that with the confounders and TMB in the model, the addition of the PHBR results in a reduction of AIC, indicating added explanatory power of PHBR even when TMB is included. In the final model with all the selected confounders, TMB and PHBR, the PHBR has a negative coefficient with a *P* value of 0.08. The AUC values associated with the final models with confounders were 0.64 for both TMB and PHBR models alone, and 0.68 for the model with both TMB and PHBR (Additional file 1: Fig. S7).

To investigate the generalizability of our analyses across histologies, we revisited Kaplan-Meier analysis for progression-free survival within tumor types with at least 5 patients (NSCLC, SCC, head and neck, breast) (Additional file 1: Fig. S8) and in all tumors excluding NSCLC and SCC, the two most common histologies (Additional file 1: Fig. S9). In each of these analyses, we observed that low versus high PHBR similarly stratified patients with high TMB. In addition, when we train a logistic regression classifier using the two most frequent histologies (*N* = 31), NSCLC and SCC, and predict response for the remaining patients (*N* = 46), we observe that the combination of PHBR and TMB better predicts OBR (Additional file 1: Fig. S10). These results suggest that the information provided by TMB and PHBR generalizes beyond high mutation burden tumors such as SCC and NSCLC.

**Fig. 2:**
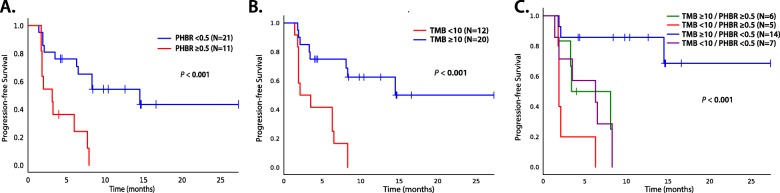
PFS for patients treated with immunotherapy in the validation dataset (*N* = 32). *P* values in the figure compare all four categories. **a** PFS dichotomized by PHBR < 0.5 and ≥ 0.5. **b** PFS dichotomized by TMB < 10 and ≥ 10 mutations/mb. **c** PFS separated by TMB < 10 and ≥ 10 and PHBR < 0.5 and ≥ 0.5

In an external validation cohort of 32 patients with NSCLC treated with pembrolizumab (Additional file 1: Table S4, Table S5 and Fig. S3), the results were similar to those in our UCSD cohort: the OBR and median PFS of PHBR < 0.5 vs. ≥ 0.5 was 76% vs. 30% (*P* = 0.02) and 14.5 vs. 2.1 months (*P* < 0.001), respectively (Fig. 2, Additional file 1: Table S6). Using a TMB cutoff of 10 mutations/mb, the median PFS of TMB high/PHBR high vs. TMB high/PHBR low was 8.1 months, versus not reached, respectively (*P* = 0.02) (Fig. 2, Additional file 1: Table S7). OS data was not available for analysis.

**Fig. 3:**
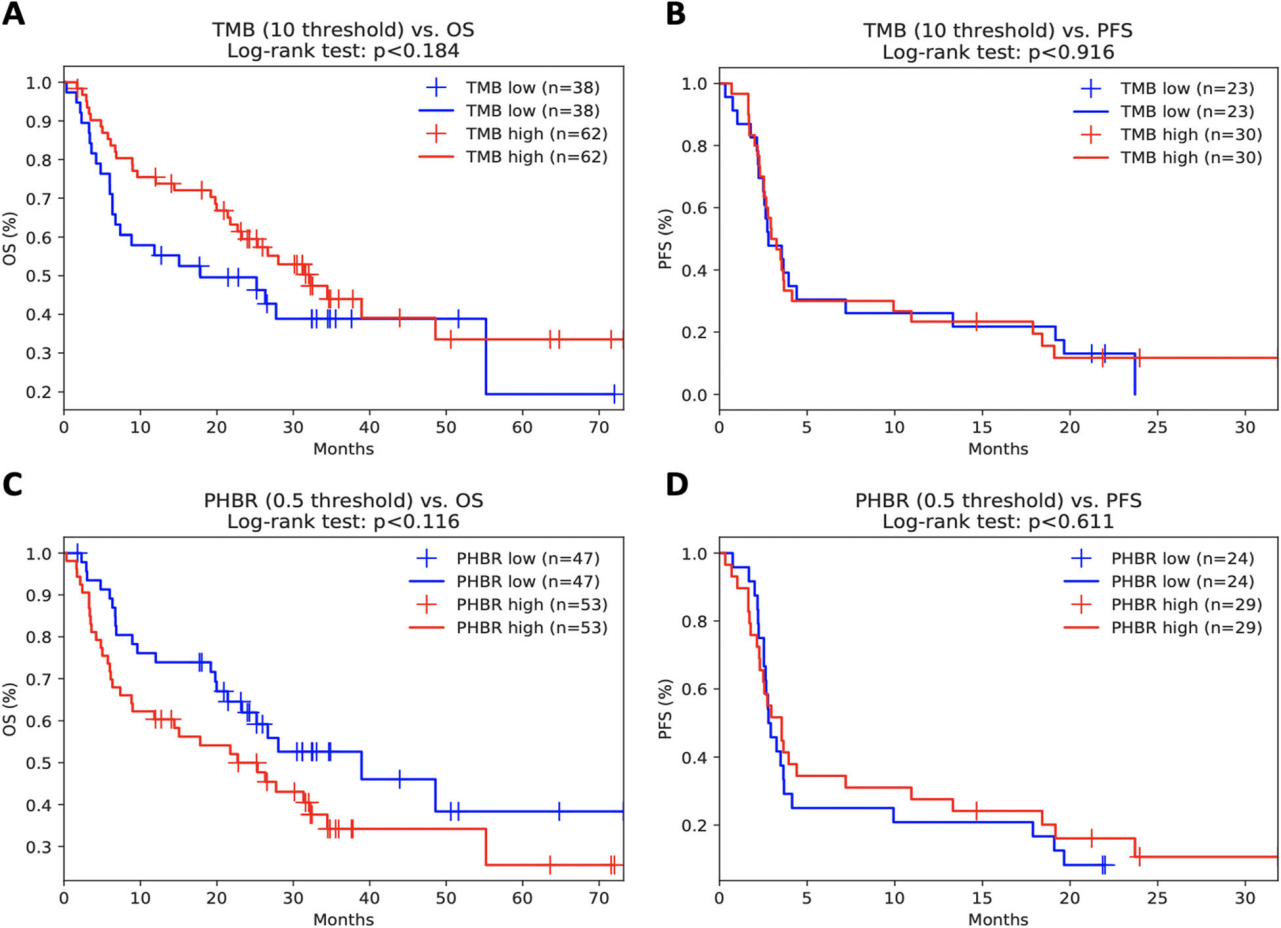
Kaplan-Meier curves showing the effects of **a** TMB on overall survival, **b** TMB on progression-free survival, and **c** minimum PHBR score on overall survival and **d** minimum PHBR score on progression-free survival in the combined melanoma cohort

**Fig. 4:**
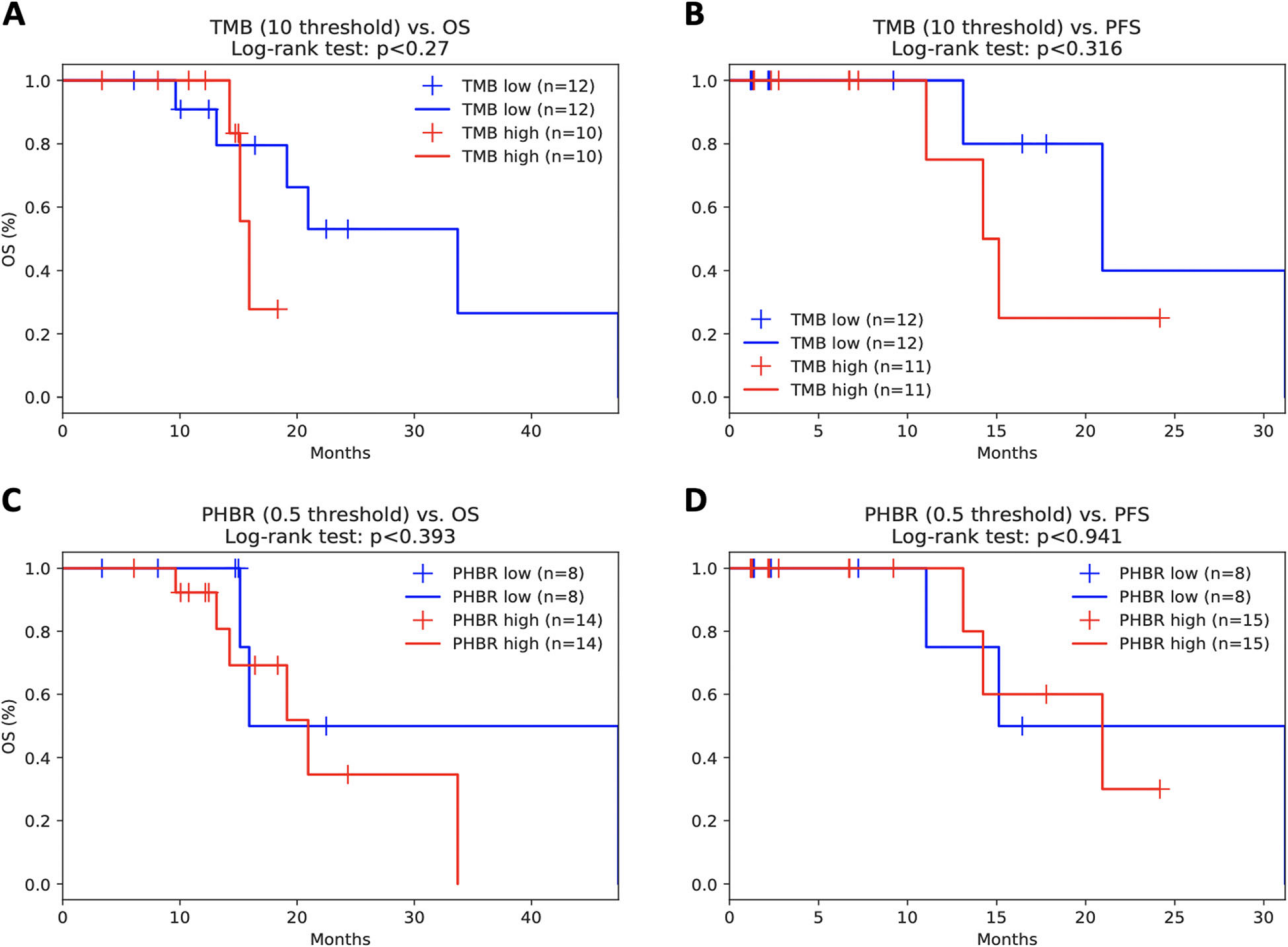
Kaplan-Meier curves showing the effects of **a** TMB on overall survival, **b** TMB on progression-free survival, and **c** minimum PHBR score on overall survival and **d** progression-free survival in the Miao kidney cohort

**Fig. 5:**
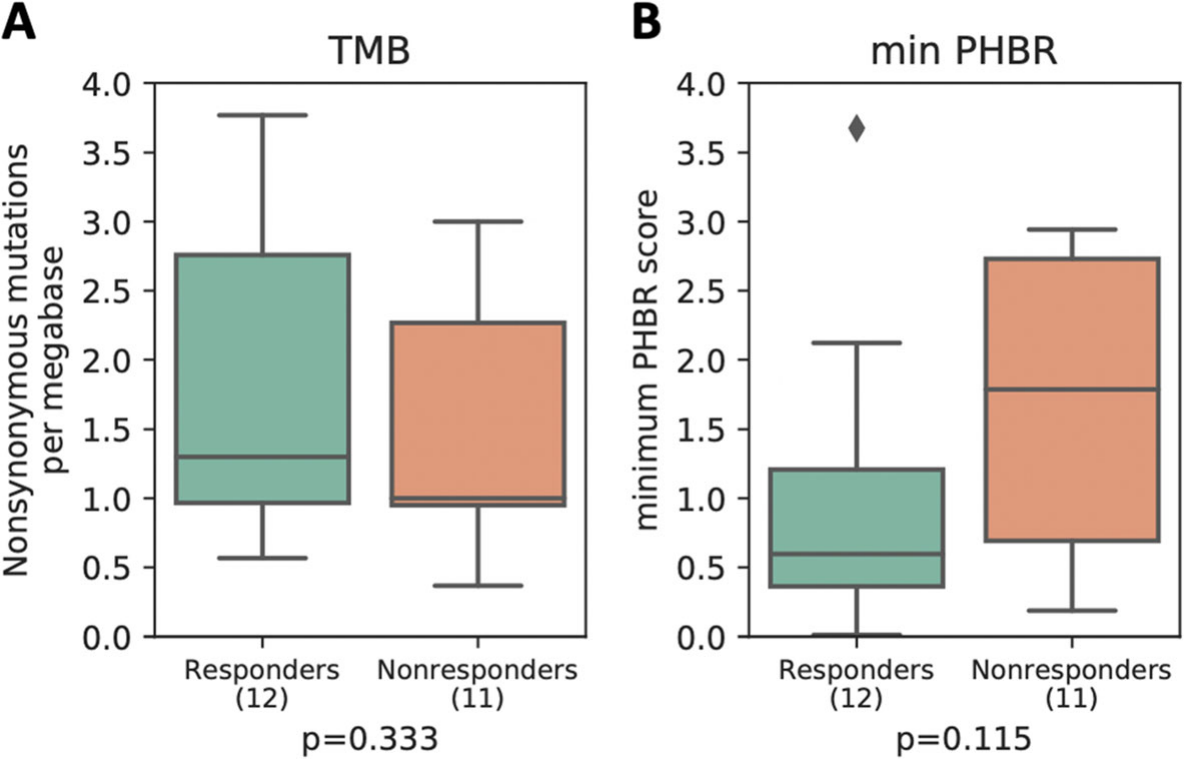
Boxplots showing the distribution of **a** TMB and **b** minimum PHBR score for responders and non-responders in the Miao cohort. *P* values were calculated by the Mann-Whitney *U* test

Finally, we compared our findings in an aggregated high-TMB melanoma cohort [44–47] and a low TMB kidney cancer cohort [48]. While minimum PHBR score did not significantly stratify melanoma patient overall or progression-free survival across all patients (Fig. 3a, b), we did find, when also considering sex and age, that lower PHBR scores (i.e., better presented mutations) were significantly associated with better overall and progression-free survival outcomes in high-TMB patients (Table 5), consistent with our reported findings. As expected in the low TMB kidney tumors, there was no correlation between mutation burden and increased progression-free or overall survival (Fig. 4a, b). Interestingly, while we did not see significant survival stratification with min-PHBR (Fig. 4c, d), we did find that responders tended to have lower PHBR scores (i.e., better presented mutations) than non-responders, although the trends did not reach statistical significance (Fig. 5).

**Table 5:** Cox proportional hazards regression for high-TMB patients in combined melanoma cohorts

Variables	Coefficients	*P* value	Confidence interval (95%)
Age	OS − 0.01	OS 0.59	OS (− 0.04, 0.02)
	PFS 0.06	PFS 0.13	PFS (− 0.02, 0.15)
Sex	OS − 0.33	OS 0.40	OS (− 1.09, 0.44)
	PFS − 0.10	PFS 0.90	PFS (−1.67, 1.47)
TMB	OS − 0.03	OS 0.05	OS (− 0.05, 0.00)
	PFS 0.03	PFS 0.24	PFS (− 0.02, 0.07)
**min-PHBR**	**OS 0.28**	OS 0.03*	**OS (0.02, 0.54)**
	**PFS 0.82**	PFS 0.02*	**PFS (0.15, 1.49)**

## Discussion

In a cohort of 83 patients with diverse solid tumors, we demonstrate that both TMB and efficient neoantigen presentation (defined by at least one PHBR score < 0.5) predict better response (as defined by SD ≥ 6 months/PR/CR rate) and longer PFS and OS after treatment with ICB. This finding was confirmed in an independent cohort of 32 patients with NSCLC treated with PD-1 blockade. Further, by incorporating the PHBR score, we were able to identify a group of higher TMB tumors (≥ 10 mutations/mb) that are less likely to benefit from ICB. Specifically, patients with tumors that poorly present driver neoantigens are less likely to respond to ICB, even in tumors with a higher mutational load. Numerous studies show that a significant proportion of patients with a higher TMB do not respond to ICB and there is a need to better identify this group of patients [15, 16, 19].

Chowell et al. demonstrated that HLA-I homozygosity and somatic loss of heterozygosity (LOH) are predictive of poor outcomes in two independent cohorts treated with ICB [49]. In addition, McGranahan et al. observed that 40% of early-stage NSCLC tumors had HLA loss of heterozygosity [32]. It was hypothesized that patients homozygous in at least one HLA-I locus would be predicted to present a smaller and less diverse tumor-derived neoantigen repertoire to CD8+ cytotoxic T cells and that the diversity of HLA molecules in a given patient influences the selection and clonal expansion of T cells following ICB [50].

Our report differs from the Chowell et al. in several ways. We assessed patient-specific MHC-I ability to bind to tumor neoantigens (PHBR score), not HLA-I diversity. Furthermore, by evaluating the interaction between TMB and the PHBR score, we demonstrated that tumors that present neoantigens efficiently respond to ICB, at least in the case of higher TMB (≥ 10 mutations/mb). However, in patients with lower TMB, the presentation of neoantigens as reflected by PHBR had no association with outcome. We hypothesize that, when there are multiple neoantigens produced by the mutanome (i.e., in patients with higher TMB), there is the opportunity for MHC-I to present them (or at least one of them) in such a way that is critical to the response. However, when there are few neoantigens, the opportunity to present them may be diminished to such an extent that the PHBR is not impactful. Additional studies will be required to better understand the neoantigen landscape as it relates to host anti-tumor immunity, in addition to the optimal method to combine information across multiple neoantigen for predicting response to therapy.

In our study, all data gathered to identify possible biomarkers to ICB was obtained via one NGS test at one time point. Prediction scores and gene signatures that take into count numerous variables including T cell infiltration into tumors, mutational load, and PD-L1 level have also been developed [51, 52]. Here we show that, with further validation, the PHBR score and TMB obtained via NGS, both of which are easy to assay, provide the ability to deliver data in real time for clinicians to make treatment decisions.

Our study has several limitations. It was a retrospective study that included a non-uniform group of patients with different malignancies treated with different checkpoint inhibitors. However, similar results were obtained in our validation cohort of NSCLC all treated with the same therapy. Our study excluded melanoma and included only small subsets of patients with individual tumor types; while our specific analyses for tumor types with ≥ 5 patients and leave-one-out analyses (Additional file 1: Fig. S8 and Fig. S9) suggest generalizability, much larger sample sizes will be required to determine whether these findings generalize to specific histologies. Our study did not assess T cell receptor (TCR) specificity and diversity. TCR specificity for MHC-I/peptide complex is essential for CD8+ T cell cellular-mediated cytotoxicity. A strong correlation between TCR CDR3 diversity and TMB has been reported [50]. Finally, we only assessed the PHBR score for MHC-I and not MHC-II. MHC-II presentation of neoantigens is possibly an important determinant of an immune response against a tumor. Frequent cancer driver mutations are poorly presented by MHC-II, and MHC-II shows less inter-patient variability but stronger selective effects than MHC-I [53].

## Conclusions

In summary, the ability of patient-specific MHC-I complexes to bind and present neoantigens represented by the PHBR score can predict who is most likely to respond to ICB within the subgroup of patients with higher TMB. These results need to be extensively validated prior to incorporation into routine clinical use. Future studies are needed to clarify the role of PHBR score in predicting response to ICB in specific malignancies. Patients with high PHBR scores may benefit from immunotherapies that circumvent antigen presentation by MHC-I (e.g., chimeric antigen receptor T cells). Finally, much effort will be needed to decipher how to best incorporate MHC-I-related PHBR, reflecting neoantigen presentation by HLA-I, in the context of PD-L1 expression, TCR repertoire, and HLA-II genotype.

## Supplementary information

**Additional file 1: Table S1.** List of patients who underwent immunotherapy at UCSD (*N* = 83). **Table S2.** Univariate analysis of factors affecting outcome for patients with TMB > 10 mutations/mb treated with immune checkpoint blockade (*N* = 39 with TMB ≥ 10 mutations/mb). **Table S3.** Multivariate analysis of factors affecting outcome for patients treated with immunotherapy (N = 39 with TMB ≥10 mutations/mb). **Table S4.** Validation cohort of 32 patients with NSCLC treated with pembrolizumab. **Table S5.** Validation cohort patient demographics by PHBR score (< 0.5 vs. ≥0.5) for 32 patients with NSCLC treated with pembrolizumab. **Table S6.** Univariate analysis of factors affecting outcome for validation patients treated with immune checkpoint blockade (*N* = 32). **Table S7.** Overall response rate and PFS, segregated by TMB low/high and PHBR low/high among validation patients (N = 32). **Table S8.** Covariates retained after the backwards selection process. The coefficients and respective *p*-values for the covariates including TMB and PHBR in the final model are shown. **Figure S1.** Overview of tumor type distribution for the discovery cohort. **Figure S2.** CONSORT Diagram. **Figure S3.** Overview of minimum PHBR score distribution and TMB distribution for the discovery (A-B) and validation (C-D) cohorts. **Figure S4.** Kaplan and Meier PFS and OS for patients treated with immunotherapy, excluding patients with TMB = 0. **Figure S5.** Additional PFS and OS for patients treated with immunotherapy (*N* = 77 with TMB available. **Figure S6.** Correlation between PHBR score and TMB (N = 77 with TMB available. **Figure S7.** Area under the receiver operating characteristic curve (AUROC) for predicting OBR in the discovery cohort using the covariates obtained from the backward selection process, with the addition of PHBR (A), TMB (B) and the combination of PHBR and TMB (C). **Figure S8.** Kaplan Meier PFS dichotomized by both PHBR < 0.5 and ≥ 0.5 and TMB < 10 and ≥ 10 mutations/mb for histologies with ≥5 patients; NSCLC (A), SCC (B), Head and Neck (C), and Breast (D). **Figure S9.** Kaplan Meier PFS dichotomized by both PHBR < 0.5 and ≥ 0.5 and TMB < 10 and ≥ 10 mutations/mb excluding NSCLC (A), SCC (B), Head and Neck (C), Breast (D) and both NSCLC and SCC, the most common histologies in our cohort (E). **Fig. S10:** Area under the receiver operating characteristic curve (AUROC) for predicting OBR from PHBR and TMB in the discovery cohort training on NSCLC and SCC patients (A) and testing on patients in the remaining tumor types (B).

## Data Availability

Clinical data used in this study is included in the online supplement. Whole exome sequencing data used in the validation analyses was downloaded from dbGaP (accession numbers: phs000980.v1.p1.c1 [40], phs001041.v1.p1 [45], phs000452.v2.p1 [46]) and the SRA (project numbers: SRP095809 [44], SRP090294 [47]).
