# Supplementary material for: MHC-I genotype and tumor mutational burden predict response to immunotherapy
Source: Genome Med. 2020 May 19;12:45. doi: 10.1186/s13073-020-00743-4 (PMC7236948; doi:10.1186/s13073-020-00743-4)
Supplement: Supplementary file 1 — Additional file 1: Table S1. List of patients who underwent immunotherapy at UCSD (N = 83). Table S2. Univariate analysis of factors affecting outcome for patients with TMB > 10 mutations/mb treated with immune checkpoint blockade (N = 39 with TMB ≥ 10 mutations/mb). Table S3. Multivariate analysis of factors affecting outcome for patients treated with immunotherapy (N = 39 with TMB ≥10 mutations/mb). Table S4. Validation cohort of 32 patients with NSCLC treated with pembrolizumab. Table S5. Validation cohort patient demographics by PHBR score (< 0.5 vs. ≥0.5) for 32 patients with NSCLC treated with pembrolizumab. Table S6. Univariate analysis of factors affecting outcome for validation patients treated with immune checkpoint blockade (N = 32). Table S7. Overall response rate and PFS, segregated by TMB low/high and PHBR low/high among validation patients (N = 32). Table S8. Covariates retained after the backwards selection process. The coefficients and respective p-values for the covariates including TMB and PHBR in the final model are shown. Figure S1. Overview of tumor type distribution for the discovery cohort. Figure S2. CONSORT Diagram. Figure S3. Overview of minimum PHBR score distribution and TMB distribution for the discovery (A-B) and validation (C-D) cohorts. Figure S4. Kaplan and Meier PFS and OS for patients treated with immunotherapy, excluding patients with TMB = 0. Figure S5. Additional PFS and OS for patients treated with immunotherapy (N = 77 with TMB available. Figure S6. Correlation between PHBR score and TMB (N = 77 with TMB available. Figure S7. Area under the receiver operating characteristic curve (AUROC) for predicting OBR in the discovery cohort using the covariates obtained from the backward selection process, with the addition of PHBR (A), TMB (B) and the combination of PHBR and TMB (C). Figure S8. Kaplan Meier PFS dichotomized by both PHBR < 0.5 and ≥ 0.5 and TMB < 10 and ≥ 10 mutations/mb for histologies with ≥5 patients; NSCLC (A), SCC [file 13073_2020_743_MOESM1_ESM.docx]

**Table S1**: List of patients who underwent immunotherapy at UCSD (N=83).

| **Patient number** | **Tumor type** | **All characterized alterations in tissue DNA sequencing** | **min PHBR score** | **TMB (muts/mb)** | **MSI Status** | **Immunotherapy regimen** | **PFS (mos)** | **OS (mos)** | | **Best response** |
| --- | --- | --- | --- | --- | --- | --- | --- | --- | --- | --- |
| 1 | Non-small cell lung cancer (squamous cell carcinoma) | STK11 V236fs*51, CDK6 amplification, IDH2 R140Q, MLL2 A3051fs*20, RUNX1T1 A491T, SMARCA4 I382fs*28, TP53 P151R | 0.1410644 | 19 | MS-Stable | Nivolumab | 1.7 | 7.4 | Progressive disease | |
| 2 | Cutaneous squamous cell carcinoma | BRCA1 E1375*, FLT3 L939F, CTNNB1 S37F, NOTCH1 P1770S, Q1810*, T1997M, TP53 R342*, ASXL1 Q748*, ATRX splice site 6327-1G>A, BRD4 rearrangement intron 11, CHD2 S521*, CREBBP Q1245*, EPHA3 T215M, FAT1 I659M, KDM6A S1400*, LRP1B loss exons 12-15, MAGI2 R766*, RB1 S474N, SETD2 R1592*, SLIT2 R1454*, splice site 611+2T>A, TERT promoter -146C>T | 0.1626378 | 347 | MS-Stable | Pembrolizumab | 26.8 | 27+ | Partial response | |
| 3 | Glioblastoma | PIK3CA E365K – subclonal, PTEN G129R, PTEN P96S, TSC1 W164* – subclonal, IDH2 R172G, ACVR1B R257H – subclonal, ATR W2565*, ATRX R1739*, CDKN2A/B loss, NTRK3 I508T – subclonal, TAF1 P163L, TP53 Y205C | 0.5390611 | 54 | MS-Stable | Nivolumab, Bevacizumab | 6.3 | 8.2+ | Partial response | |
| 4 | Non-small cell lung cancer (adenocarcinoma) | CDK4 amplification-equivocal, NF1 R416, FANCC truncation exon2, TOP2A amplification-equivocal, TP53 splice site 782+1G>T, HGF R314M, KDM6A R949fs*20, KEAP1 V420fs*15 | 1.4755458 | 15 | MS-Stable | Pembrolizumab | 31.5 | 37.1 | Partial response | |
| 5 | Basal cell carcinoma | PTCH1 S181*, PTCH1 splice site 584+1G>A, KDR R1032Q, AXIN1 T60I, BAP1 K368*, CARD11 E756K, CTNNA1 R383H, SPEN R1854Q, TP53 E285K | 0.0712556 | 45 | MS-Stable | Nivolumab | 27.4+ | 27.4+ | Partial response | |
| 6 | Head & neck squamous cell carcinoma | HRAS Q61L, CDKN2A p16INK4a loss, CDKN2A p14ARF loss exons 2-3, ARID2 Q1027*, ASXL1 G710fs*15, BCORL1 Q1664*, FAT1 Q1244*, FAT1 S869*, NOTCH2 Q1392*, PMS2 V415M, TERT promoter -124C>T, | 0.1703839 | 9 | MS-Stable | Pembrolizumab | 4 | 12 | Stable disease <6months | |
| 7 | Non-small cell lung cancer (adenocarcinoma) | EGFR E746_A750del, , EGFR T790M, AKT1 amplification, CDKN2A/B loss, TP53 R248W, NFKBIA amplification, NKX2-1 amplification | 1.2625918 | 5 | MS-Stable | Nivolumab | 1.1 | 10.1+ | Progressive disease | |
| 8 | Non-small cell lung cancer (adenocarcinoma) | EGFR amplification, EGFR L858R, EGFR T790M, MITF amplification, TP53 C238_N239>*VGSDCTTIHYNYMC, FOXP1 amplification, NFKBIA amplification, NKX2-1 amplification, NOTCH2 P6fs*27 | 0.2347227 | 5 | MS-Stable | Nivolumab, Erlotinib | 1.7 | 4.9 | Progressive disease | |
| 9 | Appendix adenocarcinoma | KRAS G12D, CDK8 amplification-equivocal, GRIN2A V1018M, TP53 G245S | 0.4853115 | 5 | MS-Stable | Pembrolizumab, Trametinib | 11.1+ | 11.1+ | Stable disease ≥6months | |
| 10 | Non-small cell lung cancer (squamous cell carcinoma) and renal cell carcinoma | ARID1A Q1519fs*13, CDK8 A155V, MAGI2 E1315fs*159, MLH1 H289fs*17, NOTCH1 R1962S, SETD2 K1969fs*2, TERT promoter -124C>T, TP53 R175H, VHL L135fs*9 | 0.1334576 | 4 | MS-Stable | Nivolumab | 5.1 | 13.6+ | Stable disease <6months | |
| 11 | Endometrial stromal sarcoma | MDM2 amplification, BCOR ZC3H7B-BCOR fusion, CDKN2A p16INK4a R58*, CDKN2A p14ARF P72L, FRS2 amplification | 0.9067467 | 3.67 | MS-Stable | Nivolumab | 1.5 | 16.6 | Progressive disease | |
| 12 | Breast cancer | AKT3 amplification – equivocal, ERBB3 M91I, NF1 S883fs*20, PIK3CA M1043I, BRCA2 Q397fs*25, AURKA amplification – equivocal, GNAS amplification, MYC amplification – equivocal, ARFRP1 amplification, GATA3 D336fs*17, PREX2 I139V, ZNF217 amplification – equivocal | 0.2096472 | 14 | MS-Stable | Pembrolizumab, Olaparib | 6 | 12 | Stable disease ≥6months | |
| 13 | Colorectal adenocarcinoma | KRAS A146T, BRCA2 V220fs*4, FBXW7 S668fs*26, GNAS R201C, MSH6 F1088fs*5, MSH6 T1219I, APC R216*, APC S1282*, BCOR K839fs*17, HNF1A G292fs*25, MLL3 K2797fs*26, SOX10 R43Q, SPEN R648W, SPTA1 R2016H | 0.3304778 | unknown | not performed | Pembrolizumab | 0.9 | 0.9 | Progressive disease | |
| 14 | Head & neck squamous cell carcinoma | CD274 (PD-L1) amplification, JAK2 amplification, PIK3CA E545K, PTCH1 M1fs*81, PDCD1LG2 (PD-L2) amplification, SOX2 amplification, SUFU S79fs*5, BCL2L2 amplification – equivocal, KEAP1 R320Q, MLL3 C310S – subclonal | 0.2546144 | 9 | MS-Stable | Pembrolizumab | 4.7 | 24.7+ | Stable disease <6months | |
| 15 | Urothelial carcinoma (bladder) | RAF1 amplification, AXIN1 R146Q, BCL2L1 amplification – equivocal⧺, CRKL amplification, MCL1 amplification, MYCL1 amplification – equivocal⧺, RB1 splice site 2490-1G>A, SLIT2 D1508fs*13, TERT promoter -124C>T, TP53 G154V | 0.3230338 | 6 | MS-Stable | Atezolizumab | 4.2 | 15.4+ | Stable disease <6months | |
| 16 | Cutaneous squamous cell carcinoma | ABL2 R359P, ERBB2 S310F, FGFR1 S790*, PDGFRA E556K, ATM Q2433*, ATM R717W, GRM3 D280N, TP53 Q331*, CSF1R splice site 1511-1G>A, FAT1 R1070*, LRP1B G1055R, NOTCH1 A1967fs*14, RB1 R579*, SPTA1 R1277H, TERT promoter -124C>T | 0.07761 | 182 | MS-Stable | Pembrolizumab | 22.4+ | 22.4+ | Complete response | |
| 17 | Non-small cell lung cancer (adenocarcinoma) | BRCA2 C1809*, CCND1 amplification, NF1 Q2002*, KRAS G12C, CDKN2A p16INK4a A57fs*51, CDKN2A p14ARF P72fs*77, FGF19 amplification, FGF3 amplification, FGF4 amplification, KEAP1 Q315*, TP53 S241F | 0.8935849 | 24 | MS-Stable | Pembrolizumab | 14.1 | 40.1+ | Partial response | |
| 18 | Renal cell carcinoma | VHL S111N, BAP1 S583*, LRP1B V3125I | 0.1092129 | 1 | not performed | Nivolumab | 2.9 | 4.4 | Stable disease <6months | |
| 19 | Gastroesophageal junction adenocarcinoma | RNF43 loss exons 3-6, CDKN2A p16INK4a L78fs*41, CDKN2A p14ARF H93fs*67, CTNNA1 loss, TP53 R248Q | 0.8736123 | 8 | MS-Stable | Pembrolizumab | 11.3+ | 11.3+ | Partial response | |
| 20 | Breast cancer | ERBB2 D769H – subclonal⧺, MTOR T1834_T1837del, PIK3CA E545K, BRIP1 R798Q, ATM R3008H, BCOR S1717*, CDH1 P260L, CDKN1B splice site 476-1G>T, MAP2K4 S184L, SMAD4 E337K, TP53 E285Q, TP53 R280K, TP53 E287* | 0.0117057 | 76 | MS-Stable | Nivolumab | 12+ | 12+ | Complete response | |
| 21 | Gastroesophageal junction adenocarcinoma | ABL1 NUP214-ABL1 fusion, CCNE1 amplification, NOTCH1 R365C – subclonal, TP53 R248W | 1.4902848 | 11 | MS-Stable | Pembrolizumab | 3.9 | 6.3 | Stable disease <6months | |
| 22 | Cutaneous squamous cell carcinoma | ATR splice site 4642-1G>A, CDKN2A/B loss, MLL2 R2635Q, NOTCH1 P391S, NOTCH2 Q831*, RAC1 P29S, SF3B1 R831Q, TERT promoter -146C>T | 0.2916894 | 12 | MS-Stable | Pembrolizumab | 21.7+ | 21.7+ | Stable disease ≥6months | |
| 23 | Cutaneous squamous cell carcinoma | CDK6 R87Q, CDKN2A p16INK4a W110*, CDKN2A p14ARF G125R, NOTCH1 P391S, NOTCH1 R353C, TP53 P152L, TP53 P98L, TP53 R282W, ASXL1 I490fs*213, CHEK2 splice site 1095+1G>A | 0.2811648 | 41 | MS-Stable | Pembrolizumab | 27.7+ | 27.7+ | Partial response | |
| 24 | Cholangiocarcinoma | PTCH1 V1131M, ARID1A R1026fs*13, BAP1 D672G, CDKN2A/B loss | 0.8512654 | 7 | MS-Stable | Nivolumab, Vismodegib, Olaparib | 6.3 | 8.1 | Stable disease ≥6months | |
| 25 | Non-small cell lung cancer (adenocarcinoma) | ROS1 amplification, STK11 I79fs*84, TSC2 E929*, BRIP1 R999*, TP53 V172F, BCL6 Q481*, DAXX S499*, MLL2 splice site 5867+1G>T, SPTA1 E1816*, ZNF217 amplification, | 0.0487073 | 57 | not performed | Nivolumab, Oxaliplatin | 30.2+ | 30.2+ | Partial response | |
| 26 | Breast cancer | MYC amplification – equivocal, CDH1 truncation exon 12, CREBBP loss exons 2-31, SMARCA4 Q1480*, TP53 C238Y | 2.6729596 | 5 | MS-Stable | Pembrolizumab | 11.7+ | 11.7+ | Partial response | |
| 27 | Non-small cell lung cancer (adenocarcinoma) | RET CCDC6-RET fusion, TP53 H193R, DNMT3A splice site 2478+1G>A, LRP1B D2600Y, NFE2L2 R18Q | 0.8526676 | 3 | not performed | Nivolumab | 0.5 | 0.5 | Progressive disease | |
| 28 | Urothelial carcinoma (bladder) | ERBB2 S310Y, ATM R2443Q, CTNNB1 S33F | 0.2367796 | unknown | MSI-Unknown | Atezolizumab | 16.6+ | 16.6+ | Partial response | |
| 29 | Spleen angiosarcoma | ATRX L1751fs*4, CDKN2A/B loss, EPHA3 amplification, MLL2 S2311fs*11, NOTCH2 M1I, TET2 A1341fs*3 | 1.1983484 | 2 | not performed | Pembrolizumab, Abraxane | 6 | 10.1 | Stable disease ≥6months | |
| 30 | Cutaneous squamous cell carcinoma | NOTCH1 R365C, NOTCH1 splice site 2467_2467+1GG>AA, TP53 R273H, TP53 S241F, ASXL1 G646fs*12, CTCF T89fs*6, FAT1 L4137fs*69, FAT1 Q3524*, LRP1B Q3874*, LRP1B splice site 13248-1G>C, NOTCH2 E1259*, NOTCH2 Q1872*, SLIT2 D704N, TERT promoter -146C>T, | 0.3415765 | 66 | not performed | Pembrolizumab | 9.8 | 11.1 | Stable disease ≥6months | |
| 31 | Colorectal adenocarcinoma | KIT V50M, PDGFRA S1042L, RET R330W, KRAS G12D, ERBB2 G1189fs*9, ERBB3 V104M, TET2 splice site 3955-2A>C, ARID1A N1784fs*5, GNAS R201H, MSH6 A1320fs*5, MSH6 R248*, PMS2 D414fs*34, APC R1114*, APC T1556fs*9, ASXL1 G645fs*58, CIC P1597fs*13+, CUL3 N290fs*21, CYLD E413fs*65, DAXX R299*, EPHB1 R507C, FAM123B E283*, JAK1 P430fs*2, MAP3K1 Q320*, MLL2 A1390fs*27, MLL2 splice site 5189-2A>G, PLCG2 A308T, RAD50 K722fs*14, SETD2 S917fs*5, SOX9 P346fs*37 | 0.2561398 | 131 | MSI-High | Pembrolizumab | 24.6+ | 24.6+ | Partial response | |
| 32 | Non-small cell lung cancer (adenocarcinoma) | CD274 amplification, PDCD1LG2 amplification, BRCA1 R1835*, HGF amplification – equivocal⧺, KRAS amplification, G12F, ARID2 S4fs*1, FGF14 amplification, JAK2 amplification, RBM10 Q358fs*125, TERT promoter -124C>T, TP53 S241C, | 0.3259145 | 7 | MS-Stable | Nivolumab | 1.6 | 1.6 | Progressive disease | |
| 33 | Appendix adenocarcinoma | KRAS G12C, PIK3CA Y1021C, CSF1R A96T, GNAS R201H | 1.5787249 | 3 | MS-Stable | Pembrolizumab, Trametinib | 6.4 | 6.5+ | Stable disease ≥6months | |
| 34 | Head & neck squamous cell carcinoma | CDKN2A p16INK4a R58*, CDKN2A p14ARF P72L, EP300 D1399N, FAT1 R2726*, MLL2 E233*, MUTYH G382D, NOTCH2 Q2006*, TERT promoter -146C>T, TP53 P301Q – subclonal⧺, TP53 R196P, TP53 R280T – subclonal⧺, WT1 C350R | 0.5784736 | 25 | MS-Stable | Pembrolizumab | 2.1 | 4.3 | Progressive disease | |
| 35 | Head & neck squamous cell carcinoma | CD274 (PD-L1) amplification, PDCD1LG2 (PD-L2) amplification, CDKN2A p16INK4a R58*, CDKN2A p14ARF P72L, APC I1307K, FAT1 S1357*, JAK2 amplification, TERT promoter -124C>T, TP53 H193N, TP53 R248W | 1.6056407 | 5 | MS-Stable | Pembrolizumab | 10.7 | 12.1+ | Stable disease ≥6months | |
| 36 | Urethral squamous cell carcinoma | BRAF G606R, EGFR amplification, FGFR2 amplification – equivocal⧺, NF1 loss exons 36-57, C17orf39 amplification – equivocal⧺, FAT1 loss, MLL3 S2860*, SPTA1 R2211H, STAT4 splice site 1571-10_1578del18 | 0.0523554 | 10 | MS-Stable | Pembrolizumab | 2.8 | 23+ | Stable disease <6months | |
| 37 | Duodenal adenocarcinoma | KRAS L19F, PTEN G132V, MEN1 E466fs*70, ARID1A Q372fs*19, BCORL1 P1681fs*20, GNAS R201H, LRP1B M482I – subclonal⧺, MSH2 Q288*, RB1 V654fs*4, SMAD4 R361H, TP53 Q167* | 0.7240415 | 8 | MSI-High | Pembrolizumab | 3.5 | 4.1 | Progressive disease | |
| 38 | Non-small cell lung cancer (adenocarcinoma) | KRAS Q61K, PIK3CA E545K, PTCH1 Q938, CHEK2 Q496, KDMSC splice site 3121-1G>A, PBRM1 splice site 715-1G>A, SETD2 Q2278, TP53 R306 | 0.3286979 | 15 | MS-Stable | Nivolumab | 3.5 | 3.5 | Partial response | |
| 39 | Non-small cell lung cancer (adenocarcinoma) | MYCL1 amplification, MUTYH G382D | 0.6725969 | 5 | MS-Stable | Nivolumab, Bevacizumab | 2.3 | 7.6 | Progressive disease | |
| 40 | Non-small cell lung cancer (adenocarcinoma) | MAP2K1 K57N, MCL1 amplification – equivocal, TP53 V203L | 0.0693966 | 18 | MS-Stable | Nivolumab, Glesatinib | 21.5+ | 21.5+ | Partial response | |
| 41 | Head & neck squamous cell carcinoma | PIK3CA amplification – equivocal, PTEN Q298*, CDKN2A p16INK4a D84Y, CDKN2A p14ARF R98L, PRKCI D396E, SOX2 amplification – equivocal, TP53 Q144*, NFE2L2 W8C | 0.3329095 | 2 | MS-Stable | Pembrolizumab | 7.7 | 12.9 | Partial response | |
| 42 | Urothelial carcinoma (bladder) | FGFR3 S249C, PIK3CA E545K, CDKN2A p16INK4a R58, CDKN2A p14ARF P72L, GATA3 H435fs*12+, MLL2 L1953fs*94, MLL2 S4147fs*2, TERT promoter-139_138CC>TT | 0.9596358 | 3 | MS-Stable | Atezolizumab | 1 | 1.2 | Progressive disease | |
| 43 | Head & neck squamous cell carcinoma | FAT1 S2682, FAT1 S3830, MPL S505N, NOTCH1 loss exons3-15, TP53 G266R | 1.3628293 | 14 | MS-Stable | Pembrolizumab | 18.5+ | 18.5+ | Partial response | |
| 44 | Basal cell carcinoma | PTCH1 E684*, CD274 (PD-L1) amplification, JAK2 amplification – equivocal, PDCD1LG2 (PD-L2) amplification, TP53 R342*, CREBBP H397fs*38, KDM5A P325S, LRP1B R2553*, STAG2 Q914*, TAF1 splice site 2119-1G>A, TERT promoter -138_-139CC>TT, WT1 S461F | 0.1847731 | 90 | MS-Stable | Nivolumab | 3.7 | 5.7 | Stable disease <6months | |
| 45 | Cutaneous squamous cell carcinoma | ARID2 P994fs*16, DNMT3A R736H – subclonal⧺, FOXP1 Q17*, MSH2 E580fs*10, SMAD4 W99*, TP53 R282W – subclonal⧺ , splice site 783-2A>T | 0.2259159 | 60 | MS-Stable | Pembrolizumab | 17.5+ | 17.5+ | Stable disease ≥6months | |
| 46 | Breast cancer | PTCH1 T416S, TP53 S127Y | 0.7431305 | 6 | not performed | Pembrolizumab | 1.2 | 8.8 | Progressive disease | |
| 47 | Non-small cell lung cancer (adenocarcinoma) | BRAF K601E, NF1 splice site 6921+1G>T, PTEN splice site 493-1G>T, CDKN2A/B loss, PBRM1 N122fs*52, RBM10 E52* | 0.9850452 | 12 | MS-Stable | Nivolumab | 0.6 | 0.6 | Progressive disease | |
| 48 | Basal cell carcinoma | PTCH G854, PTCH1 splice site 2560+1G>A, TSC1 loss exons 9-23, GRIN2A S929F, MAGI2 W688, NOTCH2 R1838, RBM10 splice site 663+1G>A, TERT promoter-146C>T, TP53 Q136, TP53 R213 | 2.0199914 | 101 | not performed | Pembrolizumab | 2.5 | 2.5+ | Progressive disease | |
| 49 | Kaposi's sarcoma | KRAS Q61H, TP53 R273C | 2.3361438 | unknown | not performed | Nivolumab | 4.9+ | 4.9+ | Stable disease (but still not reached to 6 months) | |
| 50 | Head & neck squamous cell carcinoma | PIK3CA V344G, SOX9 E75K – subclonal | 0.4246539 | 7 | MS-Stable | Pembrolizumab | 4.8 | 6.8 | Stable disease <6months | |
| 51 | Cervical squamous cell carcinoma | FBXW7 R465H, CTNNB1 S37C, BCOR Q224*, BCOR Q990*, RB1 E31*, RB1 S230* | 12.339201 | 1 | MS-Stable | Pembrolizumab | 4.4 | 5.7+ | Stable disease <6months | |
| 52 | Non-small cell lung cancer (adenocarcinoma) | EGFR L858R, ATM R1907fs*22, ATM R2443Q, FAM123B Q1096* | 0.7216017 | 3 | MS-Stable | Nivolumab | 4.9 | 21.2+ | Progressive disease | |
| 53 | Esophageal squamous cell carcinoma | BRCA2 M965fs*13, BRCA2 S884*, ERBB2 I767M – subclonal, FGFR3 S249C, CDKN2A/B loss, TP53 R175H, BCORL1 Q851*, MLL3 Q2197* – subclonal, SMAD4 D351H | 1.0917708 | 20 | MS-Stable | Nivolumab | 6.4 | 17.2 | Partial response | |
| 54 | Cutaneous squamous cell carcinoma | MLH1 E594fs*23, MSH6 R248fs*8, ARID1A P1326fs*155, ARID1A Q1519fs*8, BLM N515fs*16, CDC73 E303Q, CDKN2A p16INK4a A102fs*18, CDKN2A p14ARF A117fs*18+, CHD2 K337fs*2, CREBBP L524fs*6, GRM3 S154F, LRP1B loss exons 15-25, MLL2 G5182fs*61, MLL2 Q791fs*139, NOTCH1 P422S, PBRM1 N258fs*25, PBRM1 R534*, RB1 loss exons 1-9, SPEN A2105fs*33, STAT4 splice site 129-2delA – subclonal, TP53 P153_R156>G | 0.5285124 | 107 | not performed | Pembrolizumab | 0.3 | 0.3 | Progressive disease | |
| 55 | Prostate cancer | PIK3CA E545K, MLL2 Q4872*, TP53 E258K, TP53 R248G | 0.9287017 | 36 | MS-Stable | Pembrolizumab | 6.2+ | 6.2+ | Stable disease ≥6months | |
| 56 | Esophageal squamous cell carcinoma | BCL2L2 amplification – equivocal, FAT1 R937*, ROS1 S1810F, SMARCA4 Q1236fs*51, TP53 L252P, TP53 R196*, VHL L188fs*28+ | 0.2129755 | 17 | MS-Stable | Nivolumab | 19.1+ | 19.1+ | Partial response | |
| 57 | Cutaneous squamous cell carcinoma | TSC2 P670S, ARID2 L1790*, CDKN2A p16INK4a P114L, CREBBP Q1777*, EP300 Q447*, LRP1B W3570*, NOTCH1 Q1214*, NOTCH2 splice site 2027-1G>A, TERT promoter -124C>T, TP53 H179R, TP53 R196* | 0.112659 | unknown | not performed | Pembrolizumab | 3 | 7+ | Progressive disease | |
| 58 | Rectal squamous cell carcinoma | AKT1 E17K, CYLD Q26* – subclonal⧺, LRP1B T2117fs*5, SPTA1 R374*, STAG2 Q167* | 1.361569 | 18 | MS-Stable | Pembrolizumab | 10.8+ | 10.8+ | Stable disease ≥6months | |
| 59 | Cutaneous squamous cell carcinoma | NF2 L360fs*4, CDKN2A p16INK4a Q50*, FAT1 W1345*, LRP1B W2722*, NOTCH1 Q1957*, NOTCH2 Q2452*, PREX2 M1298I, TAF1 E1774*, TERT promoter -124C>T, TP53 R248Q, TP53 splice site 376-11_380>CCT | 0.8653269 | 78.26 | MS-Stable | Pembrolizumab | 13.3+ | 13.3+ | Partial response | |
| 60 | Non-small cell lung cancer (adenocarcinoma) | BRCA2 deletion exons 11-16, TP53 G266E | 4.1615498 | 14 | not performed | Nivolumab | 25.6 | 29+ | Partial response | |
| 61 | Papillary thyroid carcinoma | TP53 C238Y, RB1 loss exons 2-17 | 2.598116 | 5 | not performed | Nivolumab | 1.8 | 14.9 | Progressive disease | |
| 62 | Non-small cell lung cancer (squamous cell carcinoma) | PIK3CA amplification, SOX2 amplification, CDKN2A/B loss, KDM6A deletion exons 5-20, LRP1B G783V, MCL1 amplification, TP53 V274L, | 1.1696483 | 12 | MS-Stable | Nivolumab | 5.8 | 17.4+ | Stable disease <6months | |
| 63 | Esophageal squamous cell carcinoma | CDKN2A/B loss, TP53 C176F, TP53 D61fs*62, EP300 P925T | 0.910165 | 6 | not performed | Nivolumab, Palbociclib | 1.7 | 4.6 | Progressive disease | |
| 64 | Non-small cell lung cancer (adenocarcinoma) | KRAS G12V, ATM E836*, MYST3 amplification – equivocal⌘, | 2.0897946 | <=1 | not performed | Nivolumab | 1.9 | 7.5 | Progressive disease | |
| 65 | Non-small cell lung cancer (adenocarcinoma) | EGFR amplification, EGFR L858R, CDK6 amplification, BCL2L2 amplification – equivocal, NFKBIA amplification – equivocal, NKX2-1 amplification – equivocal, RPTOR amplification – equivocal, TP53 P250L, TP53 V218fs*29 | 1.1502917 | 7 | MS-Stable | Nivolumab | 1.3 | 9.3 | Progressive disease | |
| 66 | Appendix adenocarcinoma | KRAS G12V, TP53 V272L | 1.9461552 | <=1 | not performed | Pembrolizumab | 1.1 | 6.3+ | Progressive disease | |
| 67 | Non-small cell lung cancer (adenocarcinoma) | KRAS G12C, CDK12 F336fs*1, LRP1B Q2449*, RBM10 K383*, TERT promoter -124C>T, | 7.2906939 | 14.37 | MS-Stable | Pembrolizumab | 6.9 | 10.1+ | Progressive disease | |
| 68 | Cutaneous squamous cell carcinoma | CCND1 amplification, FGF19 amplification, FGF3 amplification, FGF4 amplification, LRP1B splice site 13561-1G>A, PBRM1 W1365*, TET2 Q654*, TP53 R282W | 4.6665268 | 26 | MS-high | Pembrolizumab | 3.6 | 17.1 | Progressive disease | |
| 69 | Breast cancer | PTEN loss, HGF amplification, FAS loss, RB1 rearrangement intron 10, TP53 R110P | 0.309007 | 5 | not performed | Pembrolizumab, Capecitabine | 1.7 | 1.7 | Progressive disease | |
| 70 | Adrenal cortical carcinoma | FGFR4 amplification – equivocal, PDGFRB amplification – equivocal, CDK6 amplification – equivocal, CTNNB1 S33C, RICTOR amplification, CDC73 complex rearrangement, FGF10 amplification, MLL2 S2910fs*32, TERT promoter -124C>T | 6.4572228 | unknown | not performed | Nivolumab | 20.5+ | 20.5+ | Stable disease ≥6months | |
| 71 | Head & neck squamous cell carcinoma | PIK3CA E542K, ARID2 splice site 1023+1G>A, LRP1B Q3874*, TERT promoter -146C>T | 0.6819018 | 8 | MS-Stable | Nivolumab, Cabiralizumab | 12.4+ | 12.4+ | Complete response | |
| 72 | Non-small cell lung cancer (adenocarcinoma) | TP53 E346*, KEAP1 S102fs*69, SMAD4 Y353C | 7.2727663 | 18 | not performed | Nivolumab | 3 | 4.3 | Progressive disease | |
| 73 | Glioblastoma | NF1 C124fs*41, deletion exons 1-37, MYC amplification – equivocal⧺, TP53 R280T, H3F3A K28M | 1.1110504 | 3.99 | MS-Stable | Nivolumab, Bevacizumab | 5.1 | 6.1+ | Stable disease <6months | |
| 74 | Non-small cell lung cancer (adenocarcinoma) | STK11 T186fs*97, ATM L944fs*22, CDK6 amplification, HGF amplification, MYC amplification, EPHB1 amplification, MCL1 amplification, PTPN11 D61H, WT1 splice site 1099-20_1107>GTGAGG | 0.9453714 | 3.6 | MS-Stable | Durvalumab, Tremelimumab | 9.6 | 11.1 | Stable disease ≥6months | |
| 75 | Non-small cell lung cancer (adenocarcinoma) | KRAS G12C | 14.2758 | 3 | MS-Stable | Nivolumab | 1.1 | 1.1 | Progressive disease | |
| 76 | Non-small cell lung cancer (adenocarcinoma) | AKT3 amplification – equivocal, CCND1 amplification – equivocal, KRAS G12C, FGF19 amplification – equivocal, FGF4 amplification – equivocal, MCL1 amplification, MYC amplification, FGF3 amplification – equivocal | 7.5064818 | 8 | MS-Stable | Nivolumab | 4.1 | 7.3 | Stable disease <6months | |
| 77 | Non-small cell lung cancer (adenocarcinoma) | KRAS amplification, KRAS G12D, CCNE1 amplification, TP53 E339*, CUL3 G70*, FAT1 S1803*, NOTCH2 L1279fs*36, | 5.1609063 | 39 | MS-Stable | Nivolumab | 2 | 11+ | Progressive disease | |
| 78 | Non-small cell lung cancer (adenocarcinoma) | TSC1 splice site 364-1G>C, AXIN1 A526V, CCNE1 amplification – equivocal⧺, RBM10 G374fs*111, SMARCA4 truncation exon 14, TP53 C238S | 10.686144 | 6.39 | MS-Stable | Nivolumab, cabiralizumab | 9.3 | 11.3+ | Partial response | |
| 79 | Urothelial carcinoma (bladder) | PIK3CA E545K, , PIK3CA E726K, RAF1 amplification, ARID1A D2178fs*22, BCL2L1 amplification – equivocal⧺, CCNE1 amplification, CREBBP deletion exons 1-23, LRP1B duplication exons 11-23, MLL2 Q3799*, NOTCH3 E1999*, PIK3CG amplification – equivocal⧺, RBM10 splice site 1951-1G>A, TP53 S106_Y107insC | 0.0405933 | 25 | MS-Stable | Pembrolizumab | 4.1 | 13.9 | Progressive disease | |
| 80 | Head & neck squamous cell carcinoma | TERT promoter -124C>T, TP53 R282W | 11.030134 | 4 | MS-Stable | Pembrolizumab | 0.9 | 2.1 | Progressive disease | |
| 81 | Non-small cell lung cancer (squamous cell carcinoma) | MYC amplification, TP53 R280I, BCORL1 splice site 4306-2A>G, NOTCH2 Q1634* | 13.336298 | 19 | MS-Stable | Nivolumab | 1.3 | 1.3 | Progressive disease | |
| 82 | Appendix adenocarcinoma | KRAS G12D, ATM G1102*, ATM W1795*, GATA6 amplification – equivocal, GNAS Q227E | 3.1348777 | 14 | MSI-Ambiguous | Pembrolizumab, Trametinib | 5.3 | 11.3 | Stable disease <6months | |
| 83 | Breast cancer | KDR amplification, KIT amplification, PDGFRA amplification, MYC amplification, ROS1 amplification, TP53 K132N | 3.2177788 | Unknown | MSI-Unknown | Pembrolizumab | 3.2 | 5.5+ | Progressive disease | |

**Table S2:** Univariate analysis of factors affecting outcome for patients with TMB > 10 mutations/mb treated with immune checkpoint blockade (N= 39 with TMB≥10 mutations/mb).

|  | ***Rate of SD with ≥6 month/PR/CR***^1^ | | ***PFS*** | | | ***OS*** | | |
| --- | --- | --- | --- | --- | --- | --- | --- | --- |
| ***Variable*** | ***N (%)*** | ***P-value***^2^ | ***Median,***  ***months*** | ***HR (95% CI)*** | ***P- value***^3^ | ***Median, months*** | ***HR (95% CI)*** | ***P- value***^3^ |
| **Sex**  Male (N=25) vs. Female (N=14) | 16 (64%) vs. 7 (50%) | 0.50 | 25.6 vs. 4.1 | 0.57 (0.25-1.31) | 0.18 | NR (median follow up, 22.4) vs. 12.0 | 0.34 (0.12-0.92) | **0.03** |
| **Ethnicity**  Caucasian (N=36) vs. Other ^4^ (N=3) | 21 (58%) vs. 2 (67%) | >0.99 | 9.8 vs. 6.0 | 0.38 (0.11-1.32) | 0.11 | 37.1 vs. 12.0 | 0.42 (0.09-1.91) | 0.24 |
| **Age**^5^, years  <60 (N=7) vs. ≥60 (N=32) | 4 (57%) vs. 19 (59%) | >0.99 | 6.0 vs. 9.8 | 1.45 (0.54-3.90) | 0.46 | NR (median follow up, 12.0) vs. 37.1 | 0.35 (0.05-2.68) | 0.29 |
| **Tumor type**  Head and neck SCC (N=2) vs. Not (N=37)  NSCLC (N=13) vs. Not (N=26)  Cutaneous SCC (N=9) vs. Not (N=30)  Other^6^ (N=15) vs. head and neck SCC, NSCLC, and cutaneous SCC (N=24) | 1 (50%) vs. 22 (60%)  6 (46%) vs. 17 (65%)  7 (78%) vs. 16 (53%)  9 (60%) vs. 14 (58%) | >0.99  0.31  0.26  >0.99 | 2.1 vs. 6.9  5.8 vs. 9.8  26.8 vs. 6.0  6.3 vs. 9.8 | 1.00 (0.13-7.42)  1.66 (0.73-3.76)  0.50 (0.17-1.47)  1.02 (0.44-2.35) | >0.99  0.22  0.20  0.97 | 4.3 vs. 37.1  37.1 vs. NR (median follow up, 21.7)  NR (median follow up, 22.4) vs. 37.1  17.2 vs. 37.1 | 1.40 (0.18-10.66)  1.14 (0.39-3.34)  0.67 (0.19-2.40)  1.11 (0.39-3.12) | 0.75  0.81  0.54  0.85 |
| **TMB**, mutations/mb  ≥20 (N=21) vs. 10-19 (N=18)  ≥50 (N=12) vs. 10-49 (N=27) | 14 (67%) vs. 9 (50%)  9 (75%) vs. 14 (52%) | 0.34  0.29 | 14.1 vs. 5.8  26.8 vs. 6.0 | 0.71 (0.32-1.60)  0.61 (0.24-1.55) | 0.41  0.30 | NR (median follow up, 22.4) vs. 37.1  NR (median follow up, 17.5) vs. 37.1 | 0.63 (0.24-1.70)  0.59 (0.17-2.08) | 0.36  0.40 |
| **PHBR**  <0.5 (N=18) vs. ≥0.5 (N=21) | 14 (78%) vs. 9 (43%) | **0.049** | 26.8 vs. 5.8 | 0.39 (0.16-0.91) | **0.03** | NR (median follow up, 23.0) vs. 17.2 | 0.53 (0.19-1.50) | 0.23 |
| **PD-1/L1 Therapy**  Monotherapy (N=34) vs. combination (N=5) | 19 (56%) vs. 4 (80%) | 0.63 | 6.9 vs. 6.3 | 1.28 (0.38-4.29) | 0.69 | 37.1 vs. 12.0 | 1.08 (0.24-4.80) | 0.92 |

***^1^***23 patients achieved SD with ≥6 month/PR/CR***.***

^2^Calculated using Fisher’s exact test.

^3^Calculated using the Log-rank test.

^4^Other = African American (N=1), Asian (N=1), and unknown (N=1).

^5^At time of initiation of treatment with immunotherapy

^6^Other = appendix (N = 1), basal cell carcinoma (N = 3), breast cancer (N = 2), colorectal (N = 1), gastroesophageal (N = 3), glioblastoma (N = 1), prostate (N = 1), rectal squamous cell carcinoma (N = 1), urothelial (N = 1), and urethral squamous cell carcinoma (N = 1).

**Abbreviations:** HR = hazard ratio; NR = not reached to 50%; NSCLC = non-small cell lung cancer; OS = overall survival; PFS = progression free survival; PHBR = patient harmonic-mean best rank; SCC = squamous cell carcinoma; TMB = tumor mutational burden.

**Table S3:** Multivariate analysis of factors affecting outcome for patients treated with immunotherapy (N=39 with TMB ≥10 mutations/mb)

| **Variable with univariate p value of ≤0.1 were selected for multivariate analysis** | | |
| --- | --- | --- |
| ***Rate of SD with ≥6 month/PR/CR*** | | |
| ***Group*** | ***OR (95%CI)*** | ***P-value*** |
| PHBR <0.5 versus ≥0.5 | 1.81 (1.05-3.15) | **0.049** |
| ***Progression-free Survival*** | | |
| ***Group*** | ***HR (95%CI)*** | ***P-value*** |
| PHBR <0.5 versus ≥0.5 | 0.39 (0.16-0.91) | **0.03** |
| ***Overall Survival*** | | |
| ***Group*** | ***HR (95%CI)*** | ***P-value*** |
| Male versus Female | 0.34 (0.12-0.92) | **0.03** |
| **Variables with univariate p value of ≤0.2 were selected for multivariate analysis** | | |
| ***Rate of SD with ≥6 month/PR/CR*** | | |
| ***Group*** | ***OR (95%CI)*** | ***P-value*** |
| PHBR <0.5 versus ≥0.5 | 1.81 (1.05-3.15) | **0.049** |
| ***Progression-free Survival*** | | |
| ***Group*** | ***HR (95%CI)*** | ***P-value*** |
| Male versus Female | 0.74 (0.30-1.79) | 0.50 |
| Caucasian versus other | 0.38 (0.11-1.35) | 0.13 |
| Cutaneous SCC versus other | 0.90 (0.26-3.14) | 0.87 |
| PHBR <0.5 versus ≥0.5 | 0.42 (0.17-1.06) | 0.07 |
| ***Overall Survival*** | | |
| ***Group*** | ***HR (95%CI)*** | ***P-value*** |
| Male versus Female | 0.34 (0.12-0.92) | **0.03** |

**Abbreviations**: CR = complete response; HR = hazard ratio; NSCLC = non-small cell lung cancer; OR = odds ratio; PHBR = patient harmonic-mean best rank; PR = partial response; SCC = squamous cell carcinoma; SD = stable disease; TMB = tumor mutational burden.

**Table S4:** Validation cohort of 32 patients with NSCLC treated with pembrolizumab

| Patient | Histology | Prior therapy (N) | PFS (months) | Response | PFS events | PHBR score | Mutation | TMB (mutations/mb)^1^ |
| --- | --- | --- | --- | --- | --- | --- | --- | --- |
| 1 | NSCLC NOS | 1 | 8.4 | PR | 0 | 0.043972438 | M_TP53_N131Y | 57.73333333 |
| 2 | Adenocarcinoma | 3 | 14.7 | PR | 0 | 0.262693295 | M_CDKN2A_H83Y | 33.7 |
| 3 | Adenocarcinoma | 0 | 2.1 | PD | 1 | 0.092112748 | M_NFE2L2_L309F | 33.06666667 |
| 4 | Adenocarcinoma | 5 | 8.3 | SD | 1 | 0.944870709 | M_MSH2_D597V | 30.46666667 |
| 5 | Adenocarcinoma | 1 | 16.6 | PR | 0 | 0.101309538 | M_TP53_R249M | 20.73333333 |
| 6 | Adenocarcinoma | 3 | 14.6 | PR | 0 | 0.095385021 | M_SMARCA4_A1218G | 22.83333333 |
| 7 | Adenocarcinoma | 6 | 1.9 | PD | 1 | 0.182858133 | M_TET2_G1147D | 18.6 |
| 8 | SCC | 1 | 4 | SD | 0 | 0.673285573 | M_SYK_G201C | 18.4 |
| 9 | Adenocarcinoma | 3 | 14.5 | PR | 1 | 0.069293151 | M_GRM3_L827P | 16.46666667 |
| 10 | Adenocarcinoma | 0 | 8.1 | SD | 1 | 1.196205561 | M_TP53_C275F | 14.2 |
| 11 | Adenocarcinoma | 0 | 12.6 | PR | 0 | 0.330528621 | M_INPP4B_G146V | 15 |
| 12 | Adenocarcinoma | 1 | 1.8 | PD | 1 | 4.031284687 | M_PBRM1_S1237L | 13.2 |
| 13 | Adenocarcinoma | 1 | 4.2 | PR | 0 | 0.279402713 | M_DDR2_R631L | 14.7 |
| 14 | Adenocarcinoma | 0 | 8.4 | SD | 0 | 0.2932235 | M_KRAS_G12S | 11.36666667 |
| 15 | Adenocarcinoma | 6 | 9.8 | PR | 0 | 0.467650819 | M_EPHA3_D806H | 10.86666667 |
| 16 | Adenocarcinoma | 1 | 4.4 | PR | 0 | 0.08227884 | M_NTRK3_A124D | 11.4 |
| 17 | Adenocarcinoma | 0 | 10.4 | PR | 0 | 0.062851135 | M_KMT2D_R2535H | 10.16666667 |
| 18 | SCC | 2 | 27.3 | PR | 0 | 0.40323972 | M_KLHL6_A52T | 10.53333333 |
| 19 | Adenocarcinoma | 5 | 3.3 | PD | 1 | 0.492170261 | M_GSK3B_P311L | 11.36666667 |
| 20 | Adenocarcinoma | 0 | 8.3 | SD | 1 | 0.117921117 | M_AR_G351R | 9.733333333 |
| 21 | SCC | 4 | 3.4 | PD | 1 | 1.146321169 | M_TP53_S106R | 10.93333333 |
| 22 | SCC | 0 | 1.9 | PD | 1 | 0.646619596 | M_FLT3_N289I | 8.5 |
| 23 | Adenocarcinoma | 1 | 6.5 | SD | 1 | 0.119049717 | M_PPP2R1A_S122F | 8.7 |
| 24 | Adenocarcinoma | 6 | 1.9 | PD | 1 | 0.115754684 | II_BRCA1_263_TCAGAAGGA | 6.966666667 |
| 25 | Adenocarcinoma | 2 | 1.9 | PD | 1 | 2.425954385 | M_CDC73_G306V | 6.333333333 |
| 26 | Adenocarcinoma | 2 | 1.4 | PD | 1 | 0.617838998 | M_ATM_R189K | 6.633333333 |
| 27 | Adenocarcinoma | 2 | 3.5 | SD | 1 | 0.06078564 | M_NF1_R461Q | 4.833333333 |
| 28 | Adenocarcinoma | 1 | 2.1 | PD | 1 | 0.550040379 | M_MSH3_G896R | 5.566666667 |
| 29 | Adenocarcinoma | 0 | 6.3 | SD | 1 | 1.151060375 | M_KDR_E1267K | 4.666666667 |
| 30 | Adenocarcinoma | 0 | 8.3 | PR | 1 | 0.303864547 | M_DDR2_S737C | 3.133333333 |
| 31 | Adenocarcinoma | 2 | 1.8 | PD | 1 | 0.528405399 | M_MET_C1326W | 2.666666667 |
| 32 | Adenocarcinoma | 1 | 6.3 | SD | 1 | 0.114350699 | M_SPOP_R284H | 1.5 |

^1^Estimated from total exonic mutations

**Abbreviations**: F = female; M = male; NSCLC NOS = non-small cell carcinoma not otherwise specified; PHBR = patient harmonic-mean best rank; PR = partial response; PD = progressive disease; PFS = progression free survival; SCC = squamous cell carcinoma; SD = stable disease; TMB = tumor mutational burden

**Table S5**: Validation cohort patient demographics by PHBR score (<0.5 vs. ≥0.5) for 32 patients with NSCLC treated with pembrolizumab

| ***Variable*** | ***Group*** | ***N* (32)** | ***PHBR <0.5 (N = 21)*** | ***PHBR ≥0.5 (N = 11)*** | ***P-value***^1^ | ***RR (95%CI)***^2^ |
| --- | --- | --- | --- | --- | --- | --- |
| **Sex** | Male  Female | 16  16 | 9 (56%)  12 (75%) | 7 (44%)  4 (25%) | 0.46 | 0.75 (0.45-1.26) |
| **Age** (years) | <60  ≥60 | 12  20 | 6 (50%)  15 (75%) | 6 (50%)  5 (25%) | 0.25 | 0.67 (0.40-1.24) |
| **Tumor type** | Adenocarcinoma  Not | 27  5 | 19 (70%)  2 (40%) | 8 (30%)  3 (60%) | 0.31 | 1.76 (0.56-5.29) |
|  | SCC  Not | 4  28 | 1 (25%)  20 (71%) | 3 (75%)  8 (29%) | 0.11 | 0.35 (0.06-1.94) |
|  | NSCLC NOS  Not | 1  31 | 1 (100%)  20 (65%) | 0 (0%)  11 (35%) | >0.99 | 1.55 (1.19-2.01) |
| **TMB** ^3^ (mutations/mb) | <50  ≥50 | 31  1 | 20 (65%)  1 (100%) | 11 (35%)  0 (0%) | >0.99 | 0.65 (0.50-0.84) |
|  | <20  ≥20 | 26  6 | 16 (62%)  5 (83%) | 10 (38%)  1 (17%) | 0.64 | 0.74 (0.46-1.18) |
|  | <10  ≥10 | 12  20 | 6 (50%)  15 (75%) | 6 (50%)  5 (25%) | 0.25 | 0.67 (0.36-1.24) |
| **Response** | SD ≥6 months/PR ^4^  Not | 19  12 | 16 (84%)  5 (42%) | 3 (16%)  7 (58%) | **0.02** | 2.02 (1.01-4.06) |
|  | PD  Not | 11  21 | 4 (36%)  17 (81%) | 7 (64%)  4 (19%) | **0.02** | 0.45 (0.20-1.01) |

^1^Calculated using Fisher’s exact test.

^2^Relative risk for PHBR<0.5.

^3^TMB was approximated from total exonic mutation burden by dividing by 30 (30M bases in coding region/1M bases in 1 megabase)

^4^One patient had SD, but not reached to 6 months. Only 31 patients were evaluable for this comparison.

**Abbreviations:** NSCLC NOS = non-small cell lung cancer not otherwise specified; PHBR = patient harmonic-mean best rank; PD = progressive disease; PR = partial response; RR = relative risk; SD = stable disease; SCC = squamous cell carcinoma; TMB = tumor mutational burden.

**Table S6**: Univariate analysis of factors affecting outcome for validation patients treated with immune checkpoint blockade (N = 32)*

|  | ***Rate of SD with ≥6 month/PR***^1^ | | ***PFS*** | | |
| --- | --- | --- | --- | --- | --- |
| ***Variable*** | ***N (%)*** | ***P-value*** ^2^ | ***Median***  ***(months)*** | ***HR (95% CI)*** | ***P- value***^3^ |
| **Sex**  Male (N=16) vs. Female (N=16) | 6 (40%) vs. 13 (81%) | **0.03** | 2.1 vs. 8.3 | 2.02 (0.82-4.99) | 0.11 |
| **Age**, years  <60 (N=12) vs. ≥60 (N=20) | 6 (55%) vs. 13 (65%) | 0.71 | 6.3 vs. 8.3 | 1.83 (0.75-4.46) | 0.17 |
| **Tumor type**  Adenocarcinoma (N=27) vs. Not (N=5)  SCC (N=4) vs. Not (N=28)  NSCLC NOS (N=1) vs. Not (N=31) | 17 (63%) vs. 2 (50%)  1 (33%) vs. 18 (64%)  1 (100%) vs. 18 (60%) | 0.63  0.54  >0.99 | 8.1 vs. NR  3.4 vs 8.1  NR vs 6.3 | 1.69 (0.39-7.30)  0.88 (0.20-3.86)  0.05 (0.00-467.30) | 0.46  0.87  0.30 |
| **TMB** ^4^, mutations/mb  ≥50 (N=1) vs. <50 (N=31)  ≥20 (N=6) vs. <20 (N=26)  ≥10 (N=20) vs. <10 (N=12) | 1 (100%) vs. 18 (60%)  5 (83%) vs. 14 (56%)  14 (74%) vs. 5 (42%) | >0.99  0.36  0.13 | NR vs. 6.3  NR vs 6.3  14.5 vs 2.1 | 0.05 (0.00-467.30)  0.27 (0.06-1.19)  0.21 (0.08-0.54) | 0.30  0.06  <**0.001** |
| **PHBR**  <0.5 (N=21) vs. ≥0.5 (N=11) | 16 (76%) vs. 3 (30%) | **0.02** | 14.5 vs. 2.1 | 0.22 (0.09-0.57) | <**0.001** |

^*^Overall survival data not available for validation cohort

^1^19 patients achieved SD with ≥6 month/PR***.*** One patient attained ongoing SD, but has not yet reached 6 month follow up and is therefore not considered evaluable for this parameter; only 31 patients were evaluable for this comparison.

^2^Calculated using Fisher’s exact test.

^3^Calculated using the Log-rank test.

^4^TMB was approximated from total exonic mutation burden by dividing by 30 (30M bases in coding region/1M bases in 1 megabase)

**Abbreviations:** HR = hazard ratio; NR = not reached; NSCLC NOS = non-small cell lung cancer not otherwise specified; PFS = progression free survival; PHBR = patient harmonic-mean best rank; SCC = squamous cell carcinoma; TMB = tumor mutational burden.

**Table S7**: Overall response rate and PFS, segregated by TMB low/high and PHBR low/high among validation patients (N = 32).

|  | ***Rate of SD with ≥6 month/PR/CR***^1^ | | ***PFS*** | | |
| --- | --- | --- | --- | --- | --- |
| ***Group*** | ***N (%)*** | ***P-value***^2^ | ***Median***  ***(months)*** | ***HR (95% CI)*** | ***P-value***^3^ |
| **TMB / PHBR** (TMB cutoff = 10 mutations/mb)  Low / High (N=6) vs. Low / Low (N=6)  Low / High (N=6) vs. High / High (N=5)  Low / High (N=6) vs. High / Low (N=15)  Low / Low (N=6) vs. High / High (N=5)  Low / Low (N=6) vs. High / Low (N=15)  High / High (N=5) vs. High / Low (N=15) | 1 (17%) vs. 4 (67%)  1 (17%) vs. 2 (40%)  1 (17%) vs. 12 (80%)  4 (67%) vs. 2 (40%)  4 (67%) vs. 12 (80%)  2 (40%) vs. 12 (80%) | 0.24  0.50  **0.01**  >0.99  0.60  0.27 | 1.9 vs. 6.3  1.9 vs. 8.1  1.9 vs. NR  6.3 vs. 8.1  6.3 vs. NR  8.1 vs. NR | 4.42 (1.05-18.57)  4.41 (0.85-22.73)  10.75 (2.57-45.45)  0.96 (0.27-3.44)  6.17 (1.52-25.00)  5.48 (1.19-25.21) | **0.02**  **0.049**  **<0.001**  0.94  **0.003**  **0.02** |
| **TMB / PHBR** (TMB cutoff = 20 mutations/mb)  Low / High (N=10) vs. Low / Low (N=16)  Low / High (N=10) vs. High / High (N=1)  Low / High (N=10) vs. High / Low (N=5)  Low / Low (N=16) vs. High / High (N=1)  Low / Low (N=16) vs. High / Low (N=5)  High / High (N=1) vs. High / Low (N=5) | 2 (22%) vs. 12 (75%)  2 (22%) vs. 1 (100%)  2 (22%) vs. 4 (80%)  12 (75%) vs. 1 (100%)  12 (75%) vs. 4 (80%)  1 (100%) vs. 4 (80%) | **0.02**  0.30  0.09  >0.99  >0.99  >0.99 | 1.9 vs. 8.3  1.9 vs. 8.3  1.9 vs. NR  8.3 vs 8.3  8.3 vs. NR  8.3 vs. NR | 4.87 (1.68-14.14)  34.48 (0.02-∞)  10.10 (1.21-83.33)  0.76 (0.09-6.10)  4.24 (0.52-34.48)  4.47 (0.28-71.81) | **0. 001**  0.11  **0.009**  0.78  0.14  0.25 |

^1^19 patients achieved SD with ≥6 month/PR.

^2^Calculated using Fisher’s exact test.

^3^Calculated using the Log-rank test. *P*-values in Figure 2 are different as they compare all four categories at the same time.

**Abbreviations:** NR = not reached to 50%; PFS = progression free survival; PHBR = patient harmonic-mean best rank; TMB = tumor mutational burden.

**Table S8:** Covariates retained after the backwards selection process. The coefficients and respective p-values for the covariates including TMB and PHBR in the final model are shown.

| *Covariates selected by backwards selection* | *Coefficient* | *P-value* | *Confidence Interval (95%)* |
| --- | --- | --- | --- |
| Intercept | -1.29 | 0.09 | (-2.79, 0.20) |
| MSI Status (MSS) | 1.46 | 0.04 | (0.04, 2.87) |
| Ethnicity (African American) | 10.96 | 0.83 | (-89.16, 111.08) |
| Ethnicity (Hispanic) | -9.59 | 0.85 | (-106.77, 87.60) |
| Diagnosis (Adrenal cortical carcinoma) | 10.25 | 0.75 | (-53.39, 73.89) |
| Diagnosis (Colorectal adenocarcinoma) | 2.84 | 0.59 | (-7.44, 13.13) |
| Diagnosis (NSCLC) (squamous cell carcinoma) | -9.31 | 0.89 | (-141.53, 122.92) |
| Diagnosis (Skin squamous cell carcinoma) | 0.85 | 0.39 | (-1.10, 2.80) |
| Diagnosis (Spleen angiosarcoma) | 8.52 | 0.78 | (-52.56, 69.60) |
| TMB (mutations per megabase) | 0.013 | 0.25 | (-0.0091, 0.035) |
| min PHBR score | -0.31 | 0.08 | (-0.66, 0.042) |


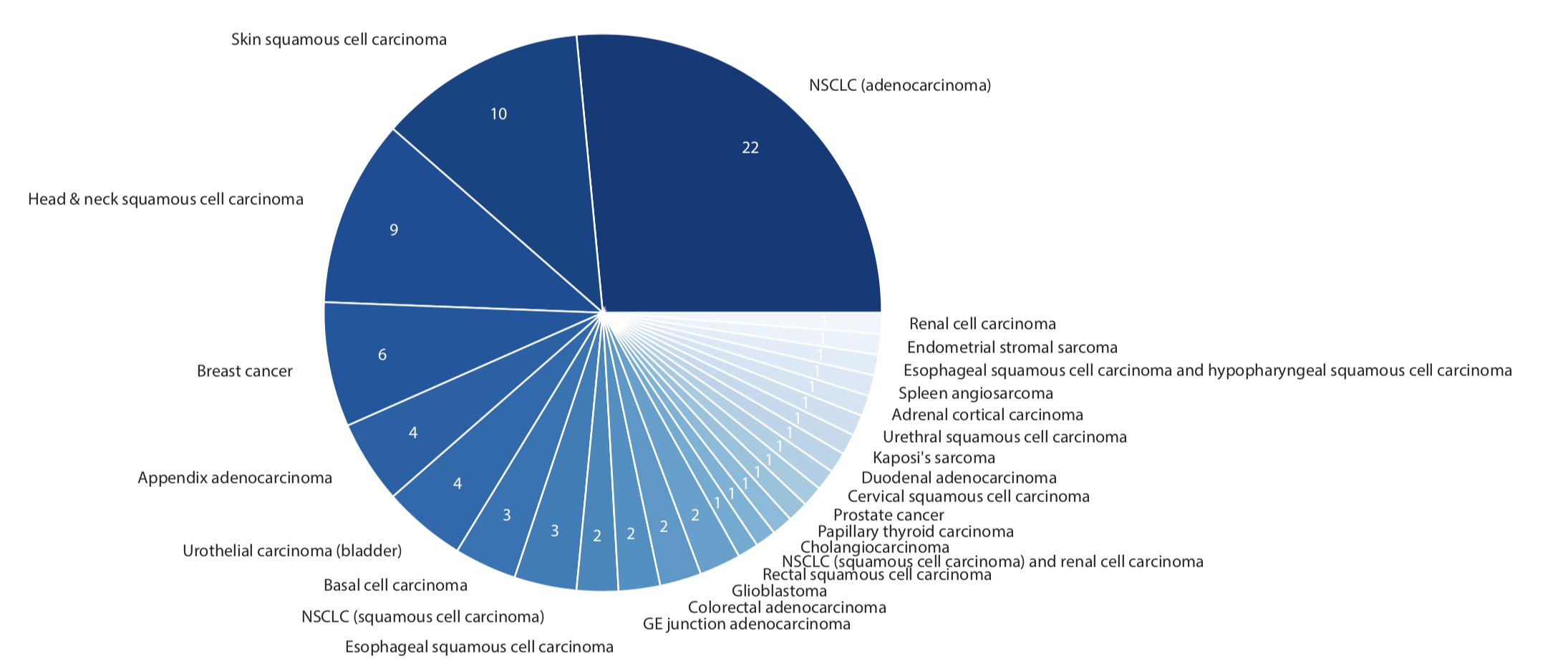


**Fig. S1:** Overview of tumor type distribution for the discovery cohort.


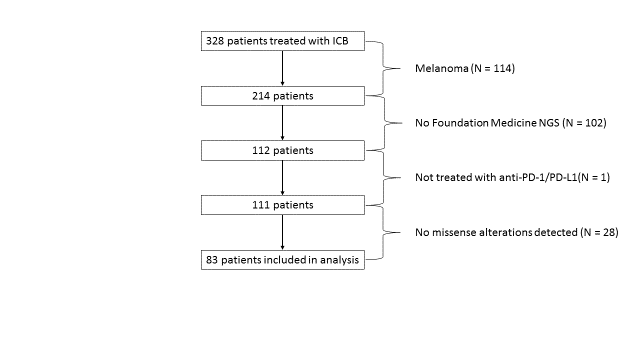


**Fig. S2**: CONSORT Diagram

**Abbreviations**: ICB = immune checkpoint blockade; NGS = next generation sequencing; PD-1/PD-L1 = programmed death receptor-(ligand) 1

**
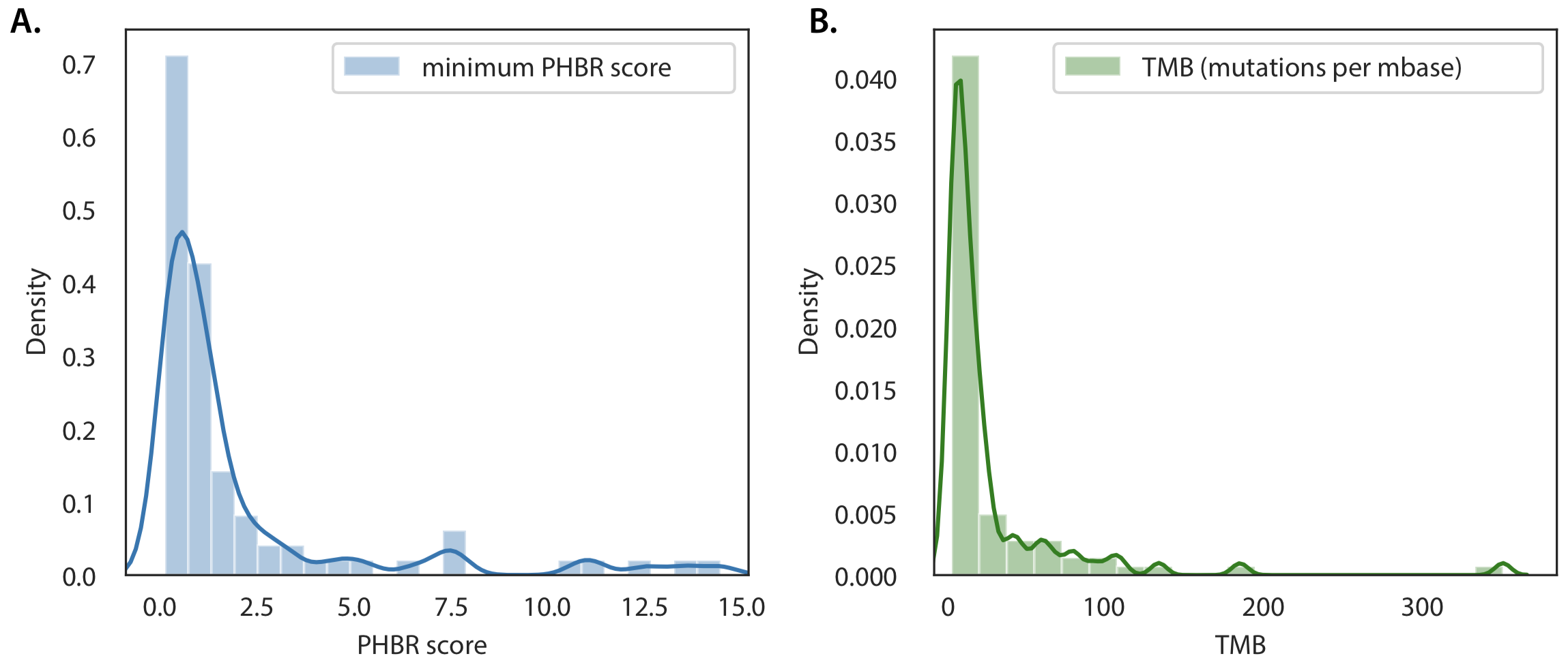
** **
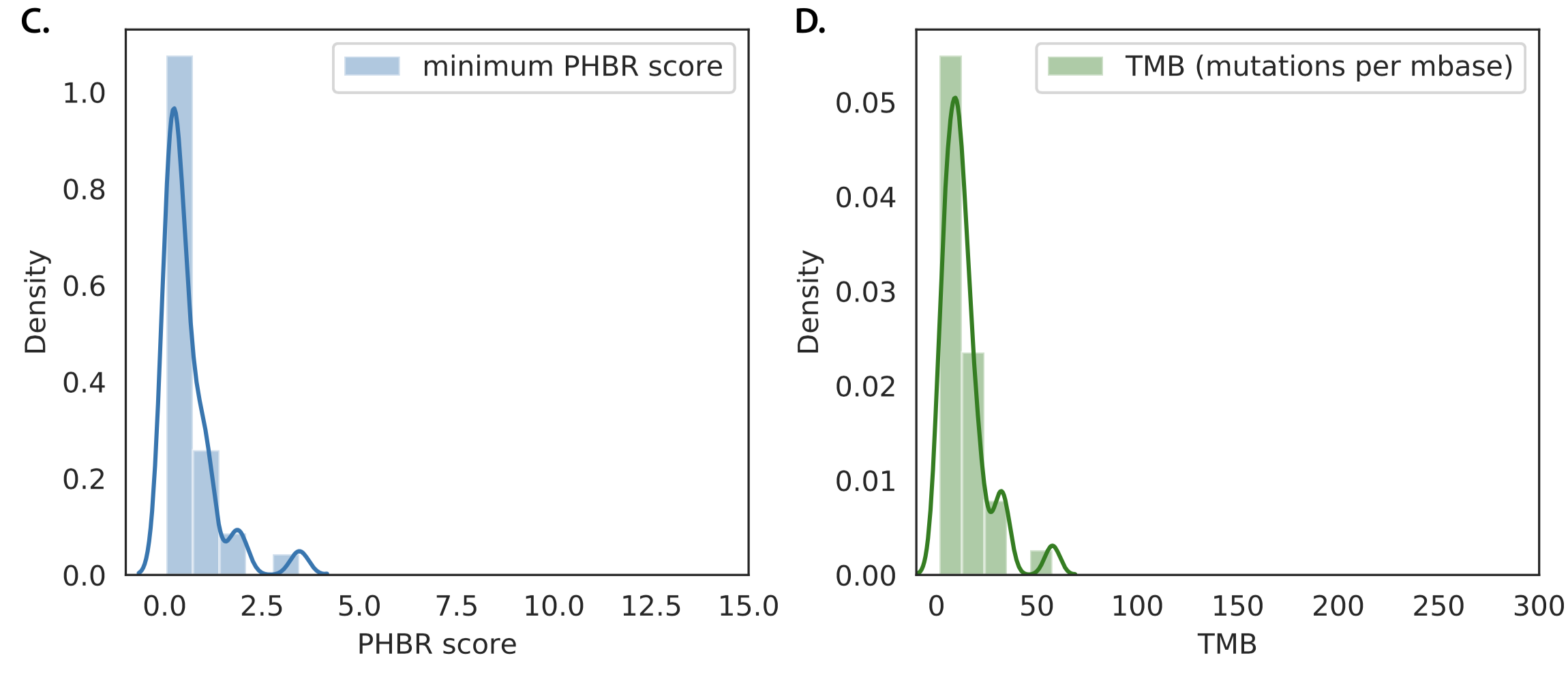
**

**Fig. S3:** Overview of minimum PHBR score distribution and TMB distribution for the discovery (A-B) and validation (C-D) cohorts.


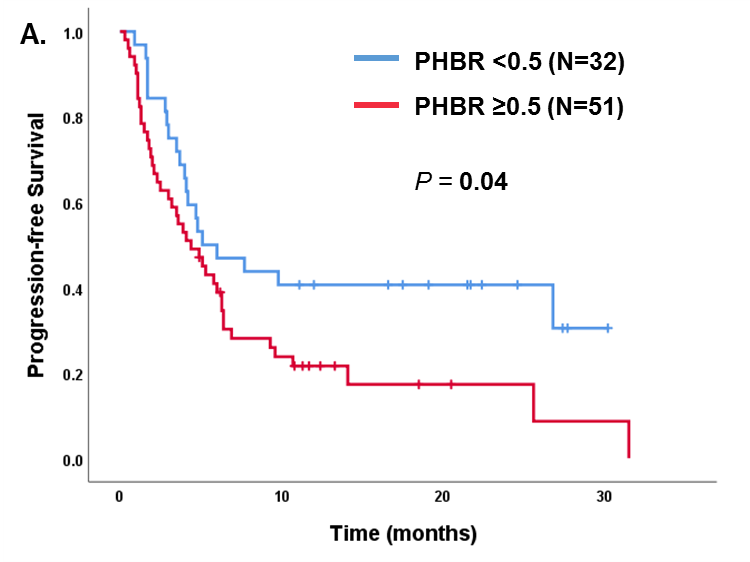

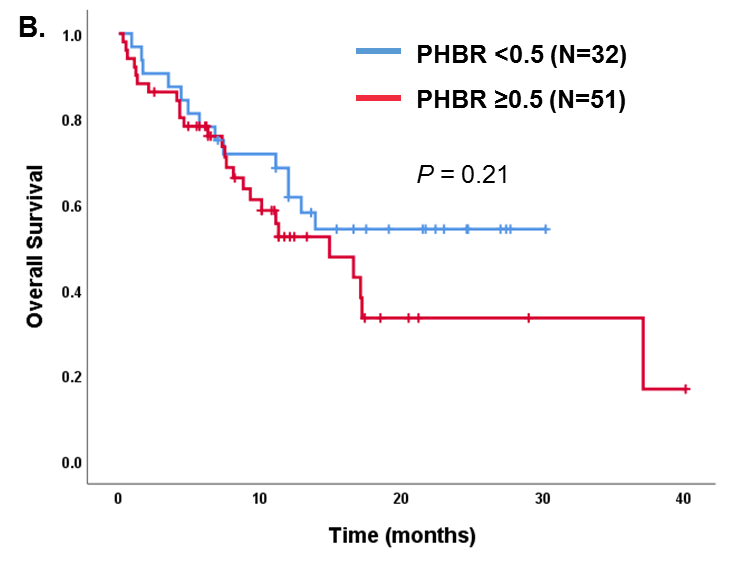

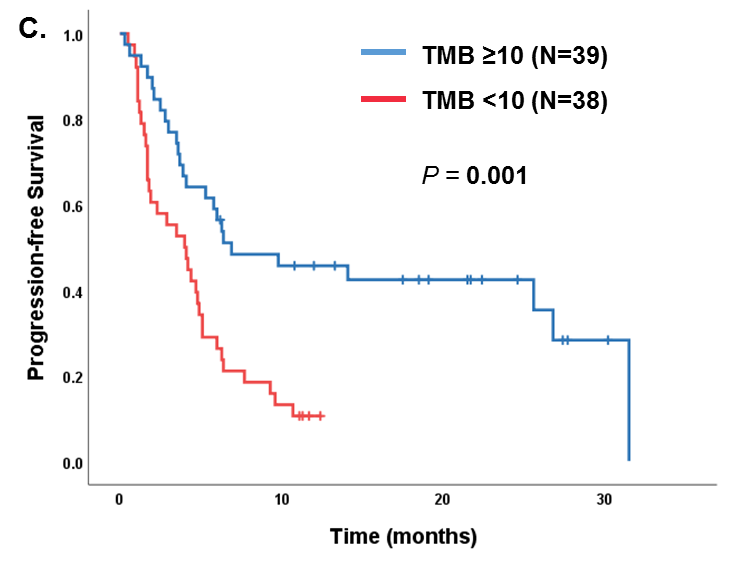

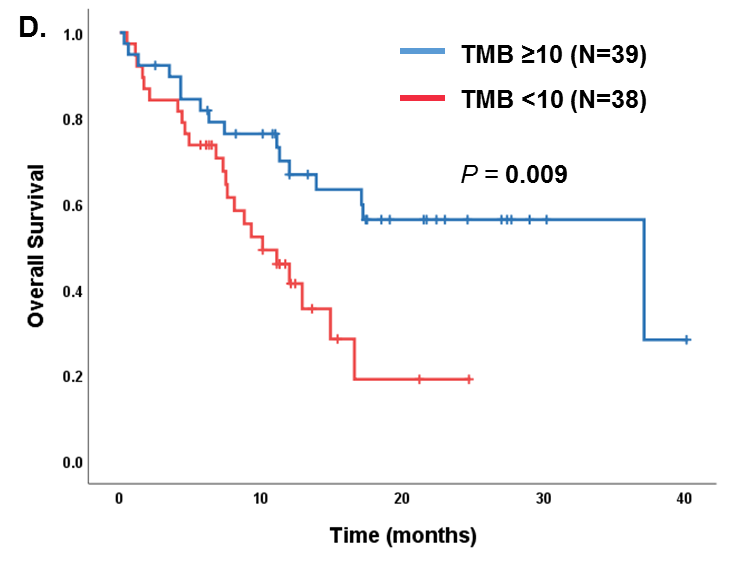

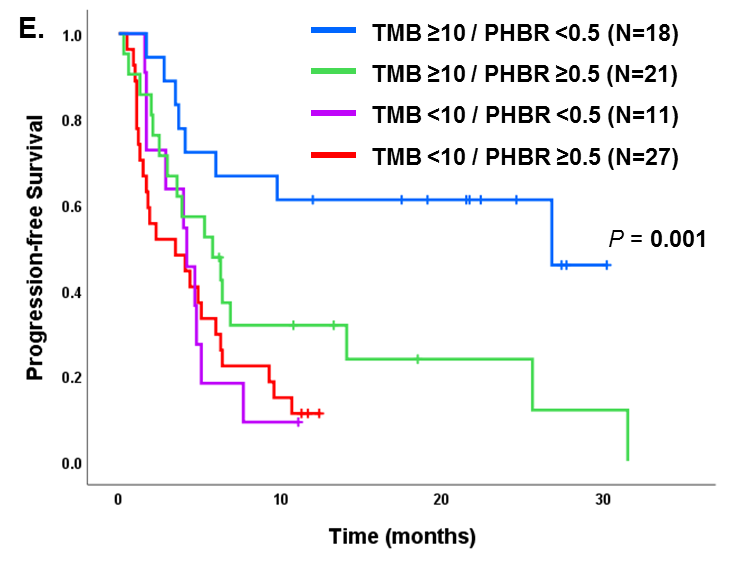

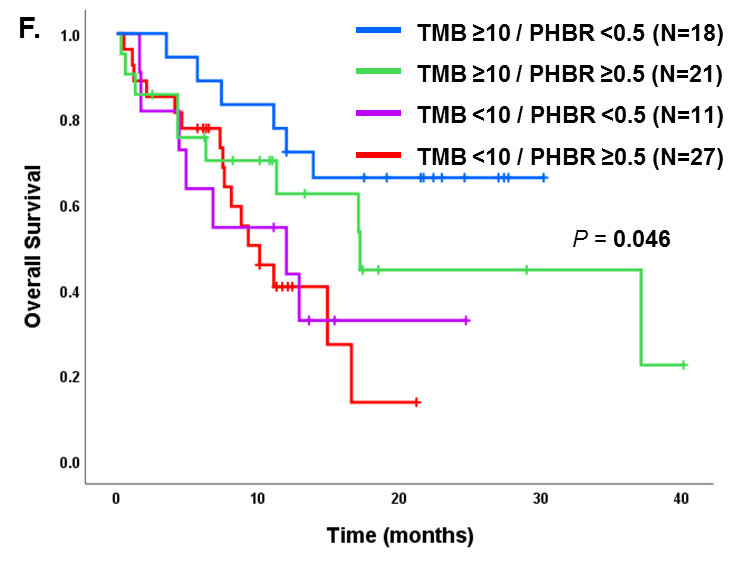


**Fig. S4:** Kaplan and Meier PFS and OS for patients treated with immunotherapy, excluding patients with TMB=0. PFS (A) and OS (B) dichotomized by PHBR <0.5 and ≥0.5 (N=83). PFS (C) and OS (D) dichotomized by TMB <10 and ≥10 mutations/mb (N=77). PFS (E) and OS (F) separated by TMB <10 and ≥10 and PHBR <0.5 and ≥0.5 (N=77 with TMB available).


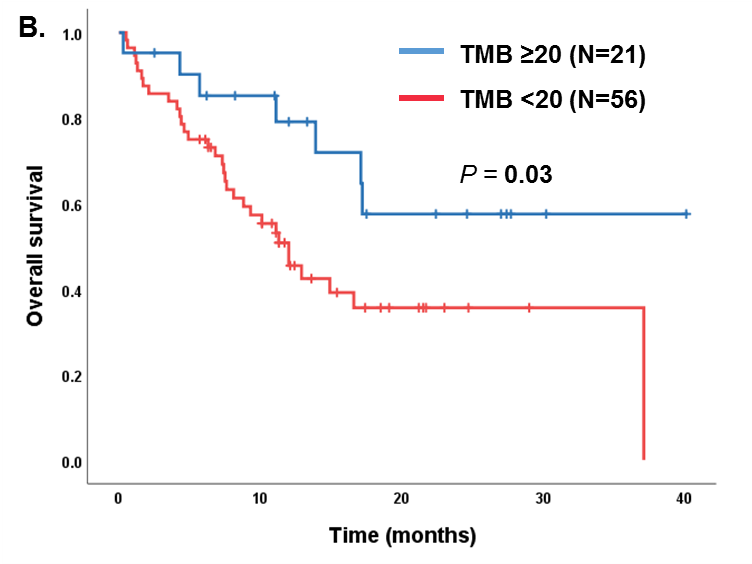

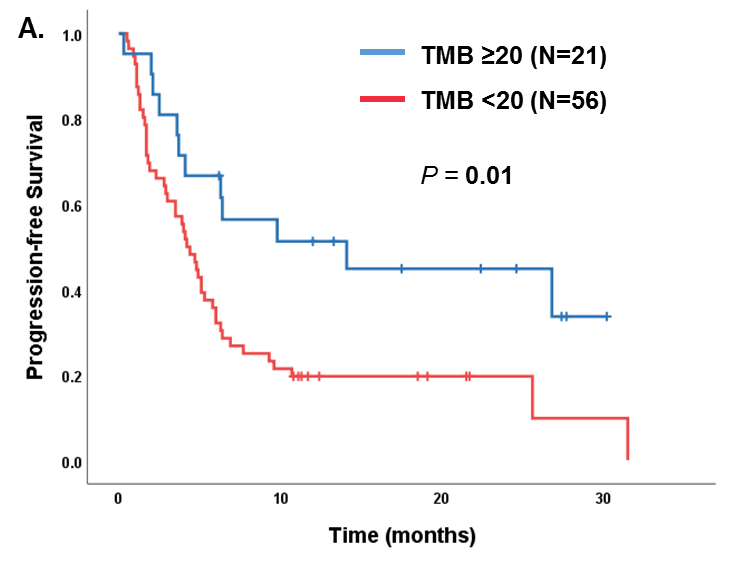


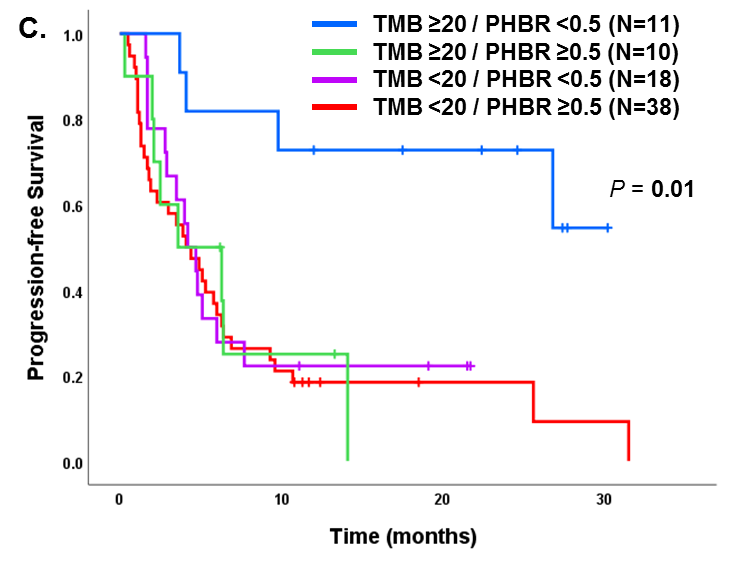

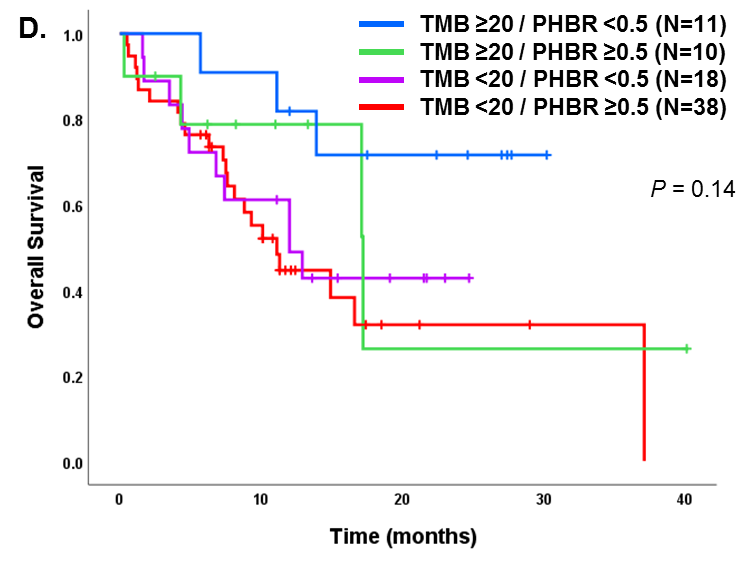


**Fig. S5:** Additional PFS and OS for patients treated with immunotherapy (N=77 with TMB available).

**Panels A and B**: PFS (A) and OS (B) dichotomized by TMB <20 and ≥20.

**Panels C and D**: PFS (C) and OS (D) separated by TMB <20 and ≥20 and PHBR <0.5 and ≥0.5


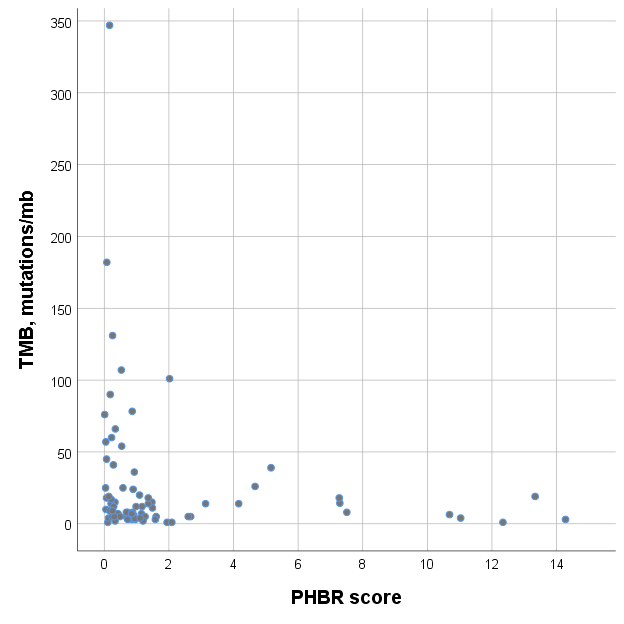


**Spearman coefficient = 0.31 (*P* = 0.01)**

**Fig. S6**: Correlation between PHBR score and TMB (N=77 with TMB available).


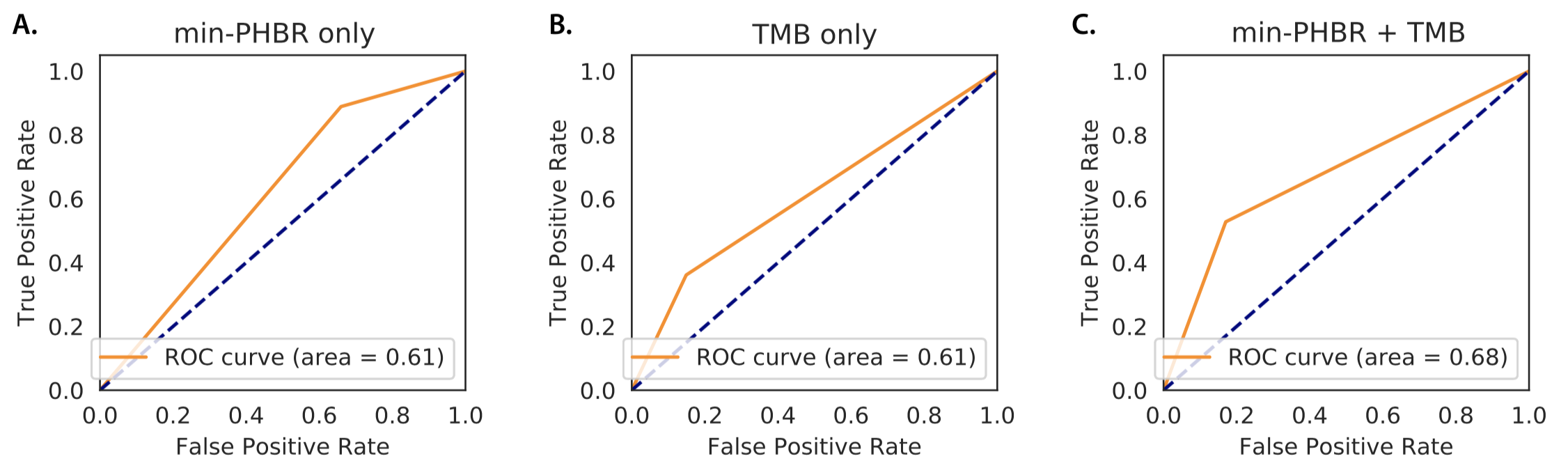


**Fig. S7:** Area under the receiver operating characteristic curve (AUROC) for predicting OBR in the discovery cohort using the covariates obtained from the backward selection process, with the addition of PHBR (A), TMB (B) and the combination of PHBR and TMB (C).

**
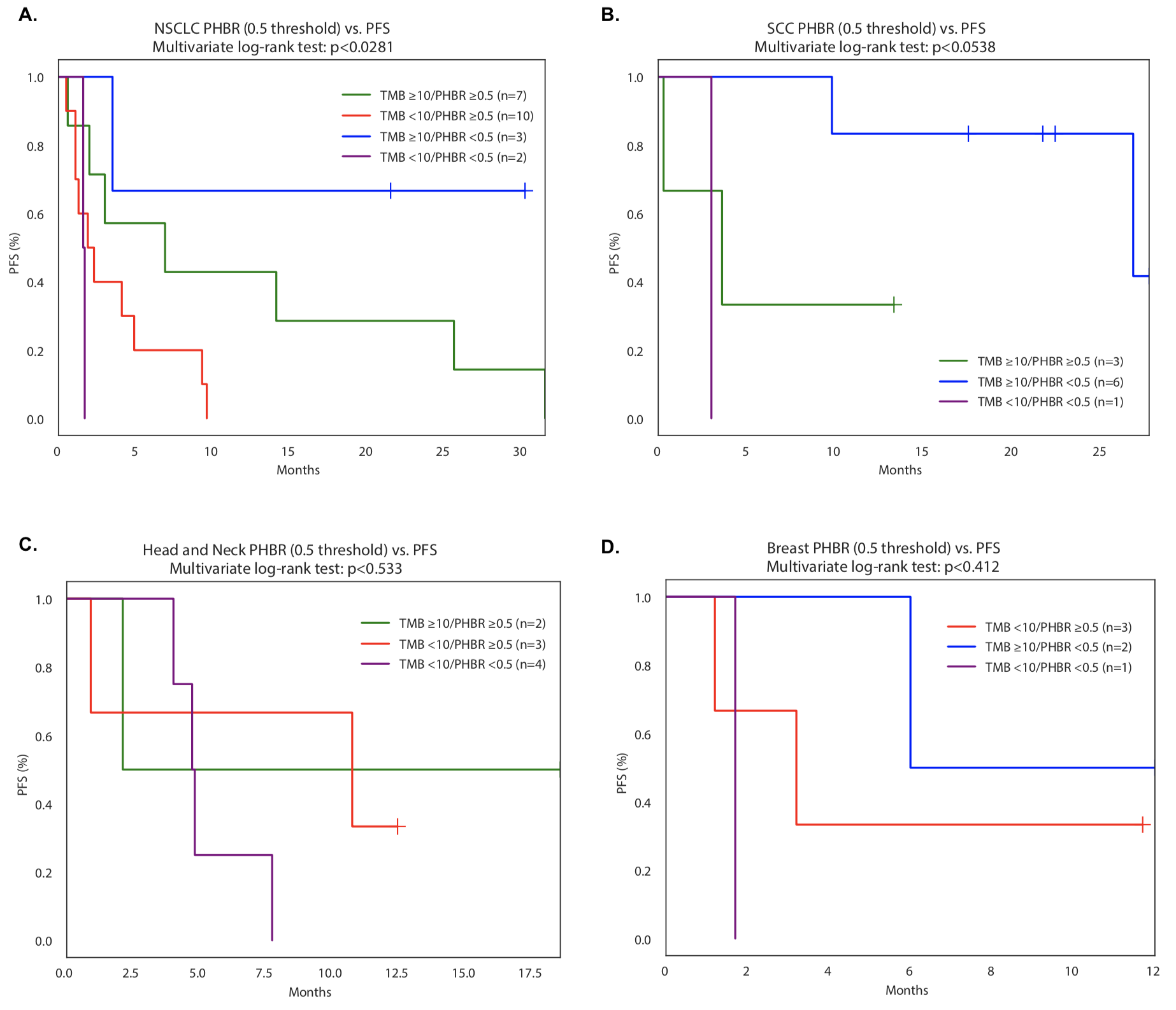
**

**Fig. S8:** Kaplan Meier PFS dichotomized by both PHBR <0.5 and ≥0.5 and TMB <10 and ≥10 mutations/mb for histologies with ≥5 patients; NSCLC (A), SCC (B), Head and Neck (C), and Breast (D).

**
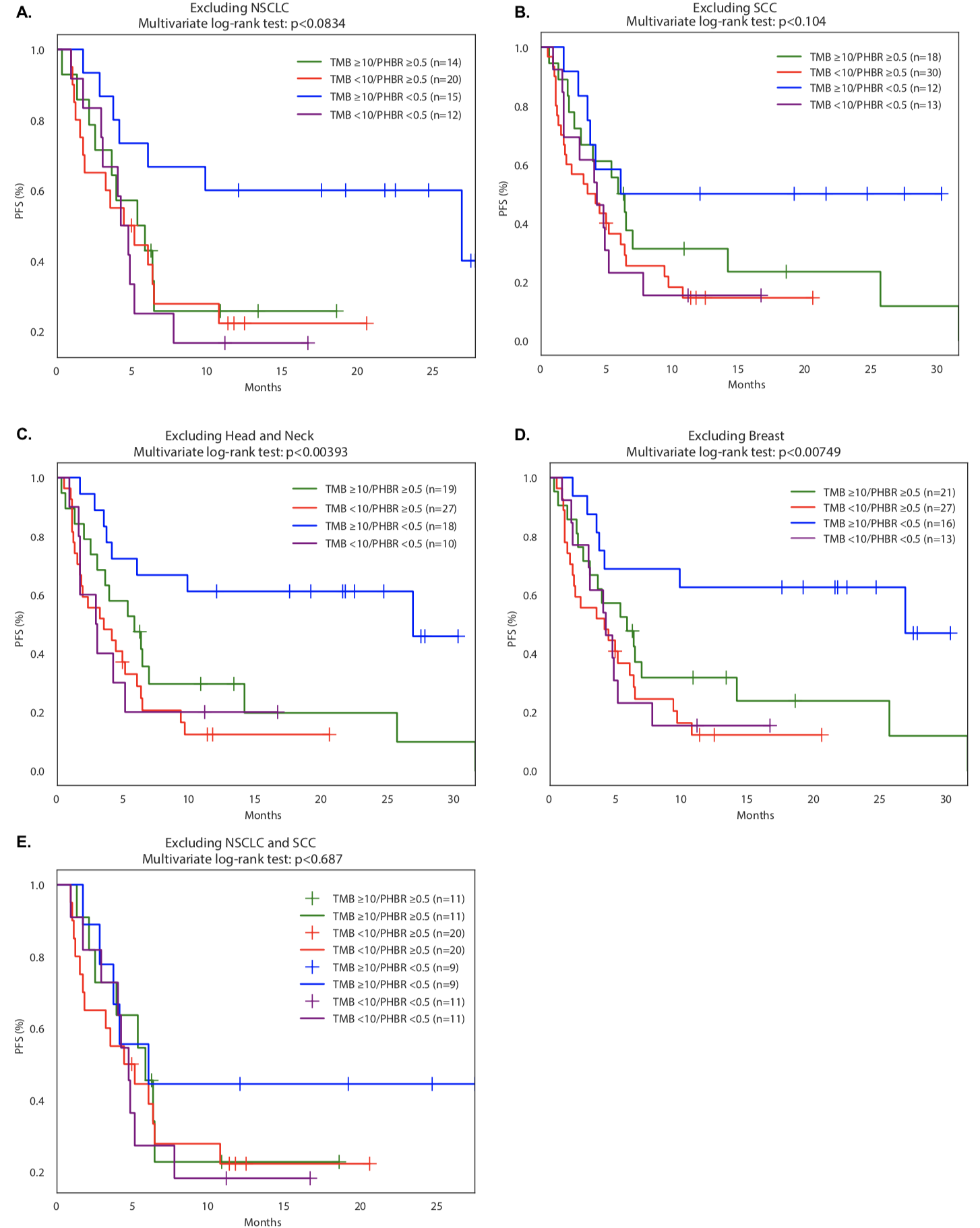
**

**Fig. S9:** Kaplan Meier PFS dichotomized by both PHBR <0.5 and ≥0.5 and TMB <10 and ≥10 mutations/mb excluding NSCLC (A), SCC (B), Head and Neck (C), Breast (D) and both NSCLC and SCC, the most common histologies in our cohort (E).


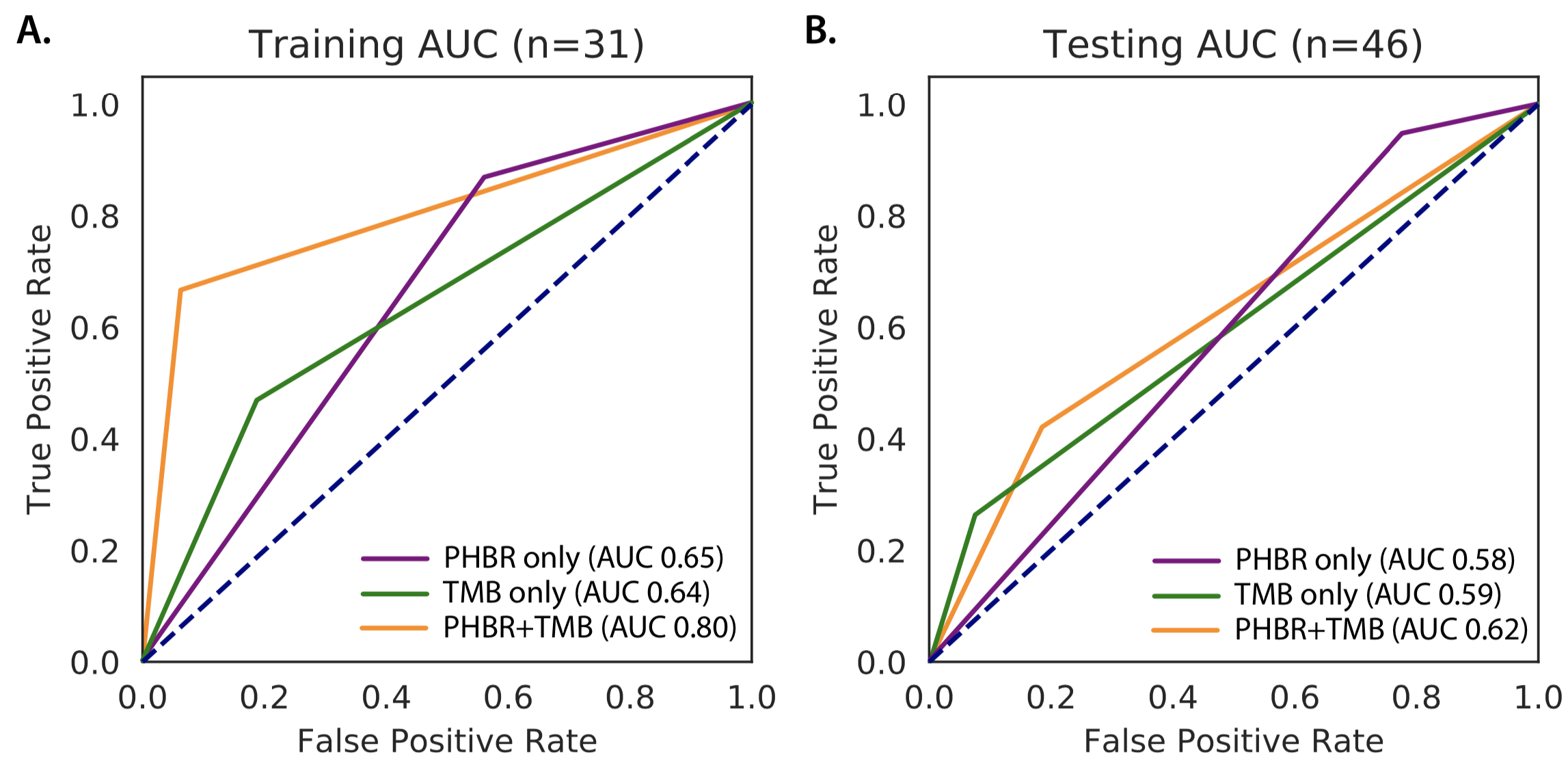


**Fig. S10:** Area under the receiver operating characteristic curve (AUROC) for predicting OBR from PHBR and TMB in the discovery cohort training on NSCLC and SCC patients (A) and testing on patients in the remaining tumor types (B).
